# Supplementary material for: X-ray-activated polymerization expanding the frontiers of deep-tissue hydrogel formation
Source: Nat Commun. 2024 Apr 15;15:3247. doi: 10.1038/s41467-024-47559-z (PMC11018743; doi:10.1038/s41467-024-47559-z)
Supplement: Supplementary file 1 — Supplementary Information [file 41467_2024_47559_MOESM1_ESM.pdf]

Supplementary Information

X-ray-activated polymerization expanding the frontiers of deep-tissue hydrogel formation

Hailei Zhang[1,*], Boyan Tang[1], Bo Zhang[1], Kai Huang[3], Shanshan Li[1], Yuangong Zhang[1], Haisong Zhang[4], Libin Bai[1], Yonggang Wu[1], Yongqiang Cheng[1], Yanmin Yang[2,*], Gang Han[3,*]

[1]College of Chemistry & Materials Science, Hebei University, Baoding 071002, P. R. China. [2]College of Physics Science and Technology, Institute of Life Science and Green Development, Hebei Key Lab of Optic-electronic Information and Materials, Hebei University, Baoding 071002, P. R. China. [3]Department of Biochemistry and Molecular Pharmacology, University of Massachusetts Medical School, Worcester, Massachusetts 01605, United States. [4]Affiliated Hospital of Hebei University, Baoding 071000, P. R. China.

*e-mail: zhanghailei@hbu.edu.cn; mihuyym@163.com; Gang.Han@umassmed.edu.

Table of Content

Supplementary Note 1: Materials....4

Supplementary Note 2: Instruments....4

Supplementary Discussion 1: Analysis for monomer conversions....5

Supplementary Discussion 2: Influence of molecular weight on the gelation behaviors....6

Supplementary Discussion 3: Study of swelling behaviors....7

Supplementary Discussion 4: Influence of X-ray irradiation time on swelling behaviors....7

Supplementary Discussion 5: Influence of the tissue depth on the radioluminescence....8

Supplementary Discussion 6: Study of swelling behaviors of the solidified hydrogel samples....8

Supplementary Discussion 7: Potential application scenarios of the proposed Xcrosslinking strategy....9

Supplementary Table 1. X-ray photoelectron spectroscopy data....11

Supplementary Table 2. Peak data in X-ray diffraction analysis....12

Supplementary Table 3. The parameters of the peaks in the X-ray diffraction patterns....14

Supplementary Figure 1. Different approaches to generate free radical polymerization via X-ray irradiation....15

Supplementary Figure 2. Photographs of the water solution containing different nanomaterials....16

Supplementary Figure 3. Nuclear magnetic resonance spectra.....17

Supplementary Figure 4. Fourier transform infrared spectroscopy results....18

Supplementary Figure 5. Thermal gravimetric results....19

Supplementary Figure 6. X-ray powder diffraction results....20

Supplementary Figure 7. Micromorphology characterizations....21

Supplementary Figure 8. Micromorphology characterizations....22

Supplementary Figure 9. Scanning transmission electron microscope-energy dispersive X-ray elemental mapping pattern....23

Supplementary Figure 10. Relationship between X-ray irradiation time and intensity of afterglow at 600 s....24

Supplementary Figure 11. Nuclear magnetic resonance spectra of photo initiators. 25
Supplementary Figure 12. Sample preparation and results in parallel experiments. 26
Supplementary Figure 13. Photographs and rheological results of the obtained hydrogels........ 27
Supplementary Figure 14. Swelling studies of dried hydrogel samples. 27
Supplementary Figure 15. Nuclear magnetic resonance results used for calculating conversion........ 28
Supplementary Figure 16. Nuclear magnetic resonance results of the samples in parallel experiments. 29
Supplementary Figure 17. Nuclear magnetic resonance results collected at different exposure times........ 31
Supplementary Figure 18. Cell viability data in HeLa cells. 33
Supplementary Figure 19. Representative photomicrograph of skin tissue sections........ 34
Supplementary Figure 20. X-ray films collected at different times exposure to Sprague Dawley rat. 35
Supplementary Figure 21. Relationship between intensity ratio and tissue thickness........ 36
Supplementary Figure 22. Swelling studies of solidified hydrogel samples. ..36
Supplementary Figure 23. Affidavit of approval of animal welfare and ethical 37

Supplementary Note 1: Materials

Halloysite nanotubes (HNTs) were obtained from GuangZhou Shinshi Metallurgy and Chemical Co., Ltd and purified from alkali water solution (pH = ca. 9, adjusted by NaOH). The HNTs-contained alkali water solution was stirred for 6 h under room temperature. Then the impurities were removed by centrifugation under 6,775 g. The supernatant is collected and further centrifugated under 9,485 rpm to afford the purified HNTs. 3-Aminopropyltriethoxysilane (KH550®), $Y(NO_3)_3 \cdot 6H_2O$, camphorquinone (CA), and poly(ethylene glycol) methyl ether acrylate (PEGMA, M. W. 480 g mol^{-1}) were purchased from Shanghai Aladdin Biochemical Technology Co., Ltd. Attapulgite nanorods (ATPs), ethylenediaminetetraacetic acid disodium salt (EDTA-2Na), and NH_4F were obtained from Shanghai Macklin Biochemical Co., Ltd. Polyethyleneglycoldiacrylate (PEGDA, molecular weight: 200, 400, and 600 g mol^{-1}) and methyl-β-cyclodextrin (MCD, degree of methyl substitution: ca. 90%) were purchased from Energy Chemical. $Tb(NO_3) \cdot 6H_2O$ was obtained from 3AMaterials®. Distilled water was used throughout the study. High-purity argon was used for degassing procedures.

Supplementary Note 2: Instruments

The X-ray-excited fluorescence emission spectra were recorded using an Andor SR-500i spectrometer (Andor Technology Co. Belfast, UK) equipped with a Hamamatsu R928 photomultiplier.

TGA was performed on a TA Q50 thermal gravimetric analyzer (TA Instruments, US) under a nitrogen flow. Accurately weighted amounts of samples were heated at a scanning rate of 10 °C min^{-1} from room temperature to 800 °C.

X-ray photoelectron spectroscopy (XPS) was carried out on a Thermo Scientific ESCALab 250Xi (Thermo Fisher Scientific, US) using 200 W monochromated Al K Alpha radiation. The 500 μm X-ray spot was used for XPS analysis. The base pressure in the analysis chamber was about 3×10^{-10} mbar. Typically the hydrocarbon C 1s line at 284.8 eV from adventitious carbon was used for energy referencing. Other acquisition parameters: Number of Scans: 5; Lens Mode: Standard; Analyser Mode: CAE: Pass Energy 30.0 eV; Energy Step Size: 0.050 eV; Number of Energy Steps: ca. 400.

Powder X-ray diffraction (PXRD) patterns were recorded on D8 ADVANCE X-ray powder diffractometer system (Bruker Corporation, German). Cu Kα source is used to generate X-ray beam with a wavelength of 1.54 Å, tube voltage of 40 kV, and current of 40 mA.

FTIR spectra were recorded in the region of 4000-400 cm^{-1} for each sample on a Nicolet iS10 Fourier transform infrared spectrophotometer (Thermo Fisher Scientific, US). Samples were previously grounded and mixed thoroughly with KBr. The spectrum for each sample was obtained from averaging 32 scans over the selected wavenumber range.

The morphological characterizations, including transmission electron

microscope (TEM) observations, energy dispersive X-ray spectroscopy (EDX) maps and high-angle annular dark-field (HAADF) Scanning TEM (STEM) images were performed by using a F200x FEI TalosF200x scanning transmission electron microscope (Thermo Fisher Scientific, US).

^{13}C solid-state nuclear magnetic resonance (NMR) spectra were obtained on a Bruker 400 M spectrometer (Bruker Corporation, German). ^{13}C NMR and ^{1}H NMR spectra were performed on a Bruker AV600 spectrometer (Bruker Corporation, German) or a QOne WNMR-I-400MHz spectrometer (Zhongke-Niujin, China) using tetramethylsilane as internal standard.

Gel permeation chromatography (GPC) (Shimazu, Japan) equipped a refractive index detector was used to determine the elution curves of the samples in parallel experiments. The column was placed under 30°C; the system was operated at a flow rate of 1.0 mL min^{-1} with tetrahydrofuran as an eluent. Polystyrene (M_p = 2,755 g mol^{-1}) was used as standards for the calibration.

AR2000ex-type rotational rheometer (TA, US) was used to investigate the rheological property.

Supplementary Discussion 1: Analysis for monomer conversions

NMR analysis was performed further to evaluate the monomer conversion at different irradiation intervals. A mono-vinyl olefin PEGMA is used to replace of PEGDA. Briefly, PEGMA, triethanolamine, complex of camphorquinone and methyl-β-cyclodextrin (MCD/CQ), and HNTs-based X-ray-activated visible persistent luminescent emitting phosphors (HNTs@YF_3:Tb^{3+}) were added into D_2O and then exposed to X-ray at different times. Other conditions are the same as the methods part in main text except for changing the irradiation times. After easing of irradiation, the samples were placed at room temperature and kept in a dark place for 24 h. Then the samples were subjected to NMR directly.

A comparison among the ^{1}H NMR spectra of PEGMA, triethanolamine, and MCD/CQ is made to pick out the peaks for the conversion calculation. As shown in Supplementary Fig. 15, the signals from the protons in vinyl groups (H_{a1}, H_{a2}, and H_b) and the proton in methylene group (H_c) that closed to ester bond do not overlap with those from triethanolamine or MCD/CQ. The vinyl groups can be consumed after the free radical polymerization of PEGMA, which would give rise to a decrease in the integral values from H_{a1}, H_{a2}, and H_b. Meanwhile, the integral value of H_c cannot changed because the methylene groups did not participate in the free radical polymerization. Therefore, the integral values from H_{a1}, H_{a2}, H_b, and H_c are used to calculate the monomer conversion (%):

$$\mathrm{Conversion}(\%) = \left[1 - \frac{(I_{\mathrm{a1}}+I_{\mathrm{a2}}+I_{\mathrm{b}})/3}{I_{\mathrm{c}}/2}\right] \times 100\% \qquad \text{Supplementary Equation (1)}$$

where I_{a1}, I_{a2}, I_b, and I_c represent the integral values from H_{a1}, H_{a2}, H_b, and

H_c respectively. The results show that the monomer conversion (%) calculated as 5.3%, 45.0%, 46.0%, and 49.0% as the irradiation time ranging from 1 min, 3 min, 5 min, and 10 min, respectively (Supplementary Fig. 17). The monomer conversion (%) increases with the increase of the irradiation time. A significant difference can be found between the monomer conversion (%) of 1 min and 3 min. After 3 min, the monomer conversion (%) changes slowly with the continuous increase of irradiation time.

Additional parallel experiments were also conducted by using PEGMA to investigate whether the polymerization behavior can take place by using these components: v: PEGMA; vi: PEGMA, MCD/CQ, and triethanolamine; vii: PEGMA and HNTs@YF_3:Tb^{3+}; viii: PEGMA, MCD/CQ, and HNTs@YF_3:Tb^{3+}. The monomer conversions (%) for the above-mentioned groups (v, vi, vii, and viii) are all calculated as ca. 0% based on the NMR results shown in Supplementary Fig. 16, indicating the polymerization cannot take place when using the above-mentioned groups containing different components. The results match well with the findings from GPC curves. The radical cannot be generated from X-ray/CQ or bypassing the composite, and the co-initiator triethanolamine is also essential for the Xcrosslinking system. Only combination of all required components, along with X-ray activation of HNTs@YF_3:Tb^{3+}, can lead to successful free radical polymerization.

Supplementary Discussion 2: Influence of molecular weight on the gelation behaviors

The impact of PEGDA's molecular weight (200, 400, and 600 g mol^{-1}) and content on gelation time and rheological properties were investigated. Here, we use $PEGDA_{200}$, $PEGDA_{400}$, and $PEGDA_{600}$ to represent PEGDA with molecular weights of 200, 400, and 600 g mol^{-1}, respectively. The contents are the same as the reaction conditions used in in vitro gelatinization study except for changing the irradiation times. $PEGDA_{200}$, $PEGDA_{400}$, and $PEGDA_{600}$ can be gelated within 60 s, 180 s, and 360 s, respectively, suggesting the gelation time increases with the increase of the PEGDA's molecular weight. However, it should be noted that $PEGDA_{200}$ cannot be dissolved in water, which yields a nonuniform hydrogel (Supplementary Fig. 13a) and is not considered for further studies. $PEGDA_{400}$ and $PEGDA_{600}$ can be dissolved in water at any proportion. The obtained $PEGDA_{400}$ and $PEGDA_{600}$ hydrogel samples (Supplementary Fig. 13b&c) were cut into desired sizes and then subjected to rheological analysis. The rheological characteristics of the resulting hydrogel were demonstrated by the storage modulus (G') being greater than the loss modulus (G'') over an average frequency range of 1 to 100 rad s^{-1} (Supplementary Fig. 13d). The $PEGDA_{600}$ hydrogel shows higher G' and G'' than the $PEGDA_{400}$ hydrogel.

The main purpose of this study is to develop an X-crosslinking approach for potential in vivo use. So, we regarded $PEGDA_{400}$ as the optimal component because it can be dissolved in water and is able to achieve a gelation state in a shorter time than that of a higher molecular weight. Furthermore, the

$PEGDA_{400}$ content (20, 30, and 40%) on gelation time was examined. $HNTs@YF_3:Tb^{3+}$ cannot achieve good dispersity when the $PEGDA_{400}$ content is higher than 50%. The results indicate that the gelation can be achieved within 600 s, 360 s, and 180 s as the $PEGDA_{400}$ content ranges from 20, 30, to 40%, respectively, implying the gelation time decreases as the increase of $PEGDA_{400}$ content and the content of 40% was used for further investigations.

Supplementary Discussion 3: Study of swelling behaviors

The swelling behavior of the synthesized hydrogels was measured by referring to the method reported by the literature.[1] The obtained hydrogel was dialyzed against deionized water thoroughly to remove the unreacted residues and then dried at room temperature. An accurately weighed dry hydrogel piece (W_0) was placed into deionized water at room temperature. The swollen samples were picked out from the medium at specific time intervals, wiped, weighed (W_i), and then placed back into the medium. The measurements were carried out in three parallel groups to calculate the mean value and standard deviation.

The swelling degree (SD_t) at various time intervals was calculated as:

$$SD_t\ (\%) = {(W_i - W_0)}/{W_0} \times 100\% \qquad \text{Supplementary Equation (2)}$$

where i represents the swollen time, W_i represents the weight of the swollen sample at different times and W_0 is the original weight of the dry hydrogel piece. The swelling behaviors were monitored until the W_i reached a constant value which is defined as the equilibrium swelling degree (ESD).

The Fickian diffusion model was employed to gain insight into the swelling kinetics.

$$\frac{M_t}{M_\infty} = \frac{SD_t}{\mathrm{ESD}} = k \times t^n \qquad \text{Supplementary Equation (3)}$$

$$\log\left(\frac{SD_t}{\mathrm{ESD}}\right) = n \log t + \log k \qquad \text{Supplementary Equation (4)}$$

where t and k represent time and a constant, respectively; M_t and M_∞ are the amount of solvent absorbed at time t (h) and at equilibrium, respectively; n is the swelling exponent, also known as the swelling exponent, is calculated based on the slope of log (SD_t/ESD) vs log$t$ (0 < SD_t/ESD ≤ 0.6).

The swelling behavior of the hydrogels was carefully investigated by plotting SD_t vs time (Supplementary Fig. 14a). SD_t increases rapidly at the first 50 min and then reaches a plateau after 120 min. The ESD is calculated as 330%. The swelling exponent (n) is calculated as 0.400, suggesting a Fickian diffusion mechanism.[2]

Supplementary Discussion 4: Influence of X-ray irradiation time on swelling behaviors

Generally, the degree of hydrogel crosslinking holds a negative correlation with swelling behaviors.[3] For the hydrogels made from the same monomers, a

higher equilibrium swelling degree (ESD) means a lower degree of hydrogel crosslinking. So, we investigated the effect of radiation time (200 s, 400 s, and 600 s) on swelling behavior in order to reveal the effect on the degree of hydrogel crosslinking. The contents and X-ray source are the same as those used in in vitro gelatinization study except for changing the irradiation times.

SD_t reaches the plateau after 120 min, 300 min, and 500 min as for the case of 600 s, 400 s, and 200 s, respectively (Supplementary Fig. 14a). The ESD values are calculated as 435 %, 352 %, and 330 % as the radiation time increases from 200 s to 600s (Supplementary Fig. 14b). The results suggest that a longer radiation time can give rise to a lower ESD value and a slower swelling behavior of the obtained hydrogel, implying the degree of hydrogel crosslinking can be well-controlled by modulating the radiation time.

Supplementary Discussion 5: Influence of the tissue depth on the radioluminescence

We have investigated the influence of the tissue depth on the radioluminescence behaviors of the prepared HNTs@YF_3:Tb^{3+}. After exposure to X-ray (40 kV, 30 mA) for 10 min, the peak intensity at 545 nm can reach a constant value and was recorded as I_0. Then the tissue samples with a certain thickness were placed between the X-ray source and HNTs@YF_3:Tb^{3+} sample. The peak intensity at 545 nm was recorded as I_t after it reached the plateau. The intensity ratio (I_t / I_0) was calculated and the relationship between intensity ratio and tissue thickness is shown in Supplementary Fig. 21. Two different kinds of tissues from chicken breast and bovine bones were used in this study.

The results indicated that the intensity ratio decreased with the increase in tissue thickness, in which the intensity ratio decreased more significantly across bone tissue than across soft tissue. We have demonstrated the gelatinization capability of our approach to enable the formation of photo-crosslinking hydrogels within thick bone tissue of ca. 7 mm. Such a high penetrability can satisfy most cases for in vivo uses. The penetrability of the proposed Xcrosslinking system is significantly higher than those of other cases including UV, visible light, and near-infrared light. A higher energy X-ray may be helpful to achieve higher penetrability.

Supplementary Discussion 6: Study of swelling behaviors of the solidified hydrogel samples

Additionally, the swelling behaviors of the original solidified hydrogel samples without the dialysis and drying procedures are also investigated. To make the experiment design align with the actual application conditions, the swelling behaviors of the hydrogel samples prepared following the on-off-on circulation process were investigated in phosphate buffer saline (PBS) of pH=7.4 at 37 °C. The accurately weighed original hydrogel sample (m_0) was directly placed into the medium. The swollen samples were picked out from the

medium at specific time intervals, wiped, weighed (m_i), and then placed back into the medium. The measurements were carried out in three parallel groups to calculate the mean value and standard deviation.

The swelling degree (SD_t*) of the original solidified hydrogel samples at various time intervals was calculated as:

$$SD_t{}^* \, (\%) = {(m_i - m_0)}/{m_0} \times 100\% \qquad \text{Supplementary Equation (5)}$$

where i represents the swollen time, m_i represents the weight of the swollen sample at different times, and m_0 is the original weight of the hydrogel sample. The swelling behaviors were monitored until the m_i reached a constant value which is defined as the equilibrium swelling degree measured from the solidified hydrogel sample (ESD*).

The swelling behaviors of hydrogel samples prepared following the on-off-on circulation process are depicted in Supplementary Fig. 22. The sample prepared after 10 cycles (1.5 mGy) shows an ESD* of ca. 48.2%, suggesting a low swelling behavior.[4-6] Moreover, the ESD* can be further decreased by extending the cycle times, which is beneficial to meet different requirements of bio-medical applications

Supplementary Discussion 7: Potential application scenarios of the proposed Xcrosslinking strategy

Visible light-cured dental resin has been commonly employed for repairing teeth defects in past decades, which affords a safer way for human bodies than ultraviolet-curing resin.[7] Though the visible light-cured dental resin can satisfy the majority of requirements for repairing teeth defects in clinical, the conventional visible light-cured strategy still suffers from the limited penetration issues in deep defects. The light intensity is distributed in a gradient decrease in the thickness direction, resulting in heterogeneity of conversion in the cured material.[8,9] As a result, the degree of conversion of the photopolymerization decreases as the increased filter thickness of dental resin filled in the defects.[10,11] For most of commercial visible light-cured dental resin, the penetration depth is usually limited to 1~3 mm.[12,13] Once the visible light-cured dental resin used to repair deep defects, the inhomogeneous irradiance may result in inadequate polymerization, shrinkage, and corresponding shrinkage stress as well as elution of low molecular substances.[14] Therefore, sometimes, visible light-cured dental resin is not recommended to be directly used in the teeth with defects exceeding 2 mm.[15,16] More operations and additional materials may be required for repairing the deep defects, which means added discomfort and a delay in patient's mouth opening time. The proposed Xcrosslinking strategy may be beneficial to break the limitation of the visible light-cured dental resin in deep defects. X-ray-activated visible persistent luminescent emitting phosphors (X-PLNPs) can serve as micro-lamps dispersed in the resin matrix rather than conventional external planar light

sources, the degree of conversion in the product afforded by the former case should not be easily affected by the thickness, and enhanced mechanical properties are also expected.[17] The Xcrosslinking strategy may be a promising supplementary technique to visible light-cured dental resin for repairing deep defects.

Supplementary Table 1. X-ray photoelectron spectroscopy data[a]

Name	HNTs		$HNTs@YF_3:Tb^{3+}$	
	Peak	Atomic %	Peak	Atomic %
Al 2p	75.19	14.71	75.07	9.74
Si 2p	103.80	16.94	103.24	13.80
C 1s[b]	285.99	1.55	286.12	5.19
O 1s	532.93	66.79	532.77	49.24
Y 3d	UN[c]	UN	160.27	3.48
Y 3s	UN	UN	396.88	3.48[d]
N 1s	UN	UN	402.39	1.41
F 1s	UN	UN	685.88	16.67
Na 1s	UN	UN	1072.96	0.28
Tb 3d	UN	UN	1277.17	0.20

[a] HNTs is abbreviated from halloysite nanotubes; $HNTs@YF_3:Tb^{3+}$ is abbreviated from halloysite nanotubes-based X-ray-activated visible persistent luminescent emitting phosphors.

[b] ~14% area is Tb 4p3

[c] “UN” means undetected

[d] repetitive computation with Y 3d

Supplementary Table 2. Peak data in X-ray diffraction analysis[a]

(h k l)	YF_3 (Standard: JCPDS[b] No. 32-1431)		Found in HNTs@YF_3:Tb^{3+}	
	2-Theta	I(f)	2-Theta	Relative intensity (0, 100)
(0 1 1)	24.058	55	23.96	31
(1 0 1)	24.626	75	24.46	73
(0 2 0)	25.986	75	25.90	97
(1 1 1)	27.884	100	27.70	100
(2 1 0)	30.96	95	30.96	83
(2 0 1)	34.784	5	34.80	7
(1 2 1)	36.07	50	35.98	21
(2 1 1)	37.232	25	37.16	12
(2 2 0)	38.575	19	38.54	12
(0 0 2)	41.068	45	40.94	13
(2 2 1)	43.915	80	43.82	35
(1 1 2)	45.618	85	45.54	22
(1 3 1)	46.993	100	46.86	55
(3 0 1)	47.568	95	47.54	50
(2 3 0)	49.041	100	48.96	60
(0 2 2)	49.239	60	49.08	40
(3 1 1)	49.496	70	49.44	27
(1 2 2)	51.42	70	51.3	15
(2 1 2)	52.309	70	52.2	22
(0 4 0)	53.408	60	53.30	18
(3 2 1)	54.989	70	54.90	26
(4 0 0)	57.897	35	57.16	4
(1 4 1)	59.64	35	59.64	8
(1 3 2)	60.166	25	60.1	5
(3 1 2)	62.281	35	62.3	7
(3 3 1)	63.394	45	63.3	13
(4 1 1)	63.593	30	63.58	8
(4 2 0)	64.506	25	64.46	8
(0 1 3)	65.103	25	65.1	7
(2 4 1)	65.328	20	65.21	8
(2 3 2)	65.827	50	65.68	7
(1 1 3)	66.94	12	66.96	4
(0 4 2)	69.51	25	69.48	4
(2 0 3)	70.789	17	70.76	6
(1 4 2)	71.295	20	71.30	5
(0 5 1)	72.088	11	72.14	4
(4 3 0)	72.292	15	72.26	4
(4 0 2)	73.42	20	73.32	6
(1 5 1)	73.847	25	73.68	6
(3 4 1)	74.273	55	74.12	11
(2 5 0)	75.414	20	75.22	5

[a] HNTs@YF_3:Tb^{3+} is abbreviated from halloysite nanotubes-based X-ray-activated visible persistent luminescent emitting phosphors.
[b] JCPDS is abbreviated from Joint Committee on Powder Diffraction Standards.

Supplementary Table 3. The parameters of the peaks in the X-ray diffraction patterns[a]

No.	2-Theta	
	HNTs	Found in $HNTs@YF_3:Tb^{3+}$
1	11.90	11.96
2	19.98	19.96
3	24.45	24.44
4	34.93	35.00
5	35.76	35.98[b]
6	38.62	38.54
7	55.00	54.90[c]
8	62.31	62.30
9	73.14	73.20

[a] HNTs is abbreviated from halloysite nanotubes; $HNTs@YF_3:Tb^{3+}$ is abbreviated from halloysite nanotubes-based X-ray-activated visible persistent luminescent emitting phosphors.

[b] overlapped with the peak of (121) in YF_3

[c] overlapped with the peak of (321) in YF_3

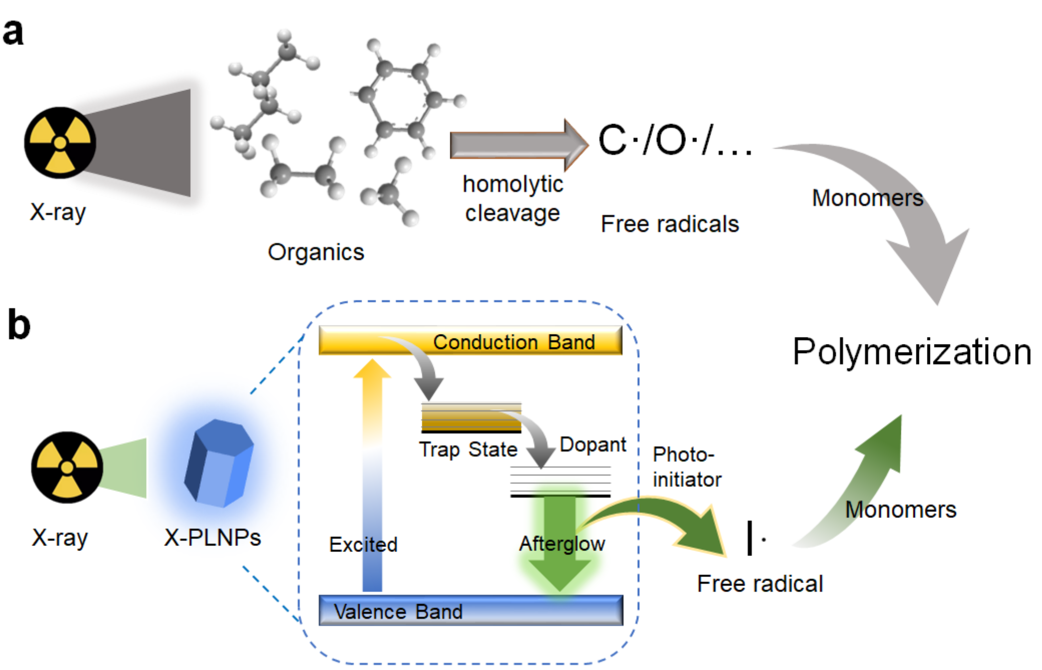

Supplementary Figure 1. Different approaches to generate free radical polymerization via X-ray irradiation. a High dose approach. In this process, the free radicals are mainly generated by the X-ray-induced homolysis reactions in which high energy is essentially needed. For example, the total dose used in the literature by using this approach is calculated as 3.3×10^3 Gy to 1.98×10^4 Gy.[18] **b** Low dose approach. The Xcrosslinking proposed in this study can be classified into a kind of cascade reaction, in which the X-ray is used to activate the luminescence and afterglow from X-ray-activated visible persistent luminescent emitting phosphors (X-PLNPs). The luminescence and afterglow can be further used to activate the photoinitiators and then generate free radicals for polymerization. X-PLNPs can be activated in a much lower X-ray dose than that used in inducing homolysis reactions.

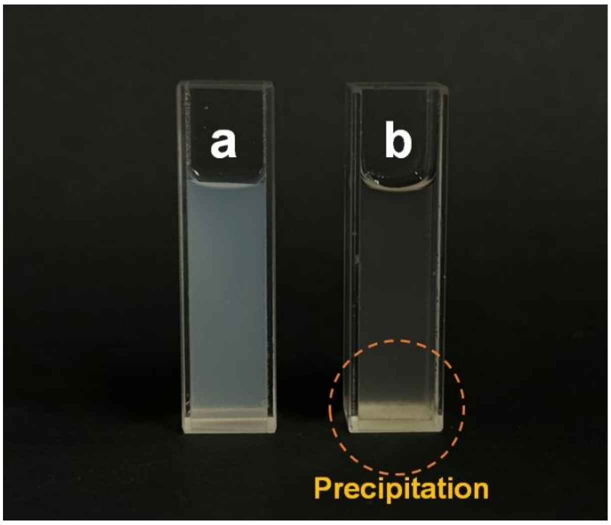

Supplementary Figure 2. Photographs of the water solution containing different nanomaterials. a Halloysite nanotubes (HNTs, concentration: ca. 1.0 mg mL^{-1}). **b** Commercially available attapulgite nanorods (ATPs, concentration: ca. 1.0 mg mL^{-1}).

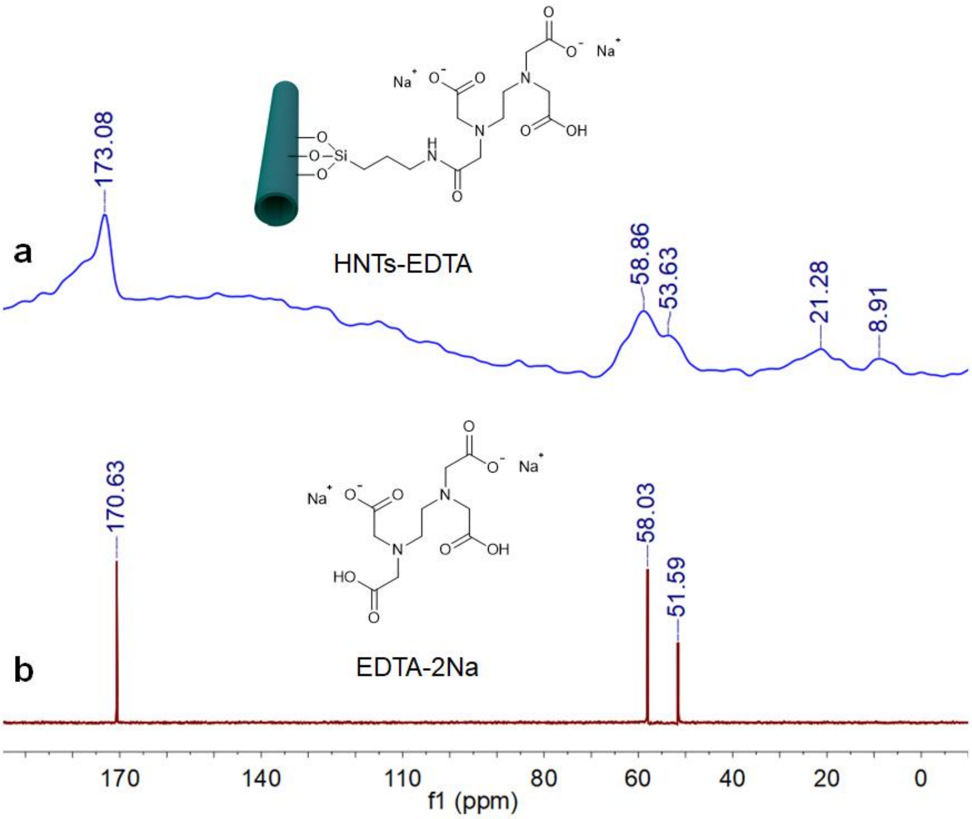

Supplementary Figure 3. Nuclear magnetic resonance spectra. a Solid-state ^{13}C nuclear magnetic resonance (NMR) spectrum of ethylenediaminetetraacetic acid-derivatized halloysite nanotubes (HNTs-EDTA). **b** ^{13}C NMR spectrum of ethylenediaminetetraacetic acid disodium salt (EDTA-2Na) measured in D_2O. The weak resonances peaked at 173.08, 58.86, and 53.63 ppm in the solid-state ^{13}C NMR spectrum of HNTs-EDTA are corresponding to carbons in C=O and $-CH_2-$ groups, respectively, as evidenced by the ^{13}C NMR spectrum of EDTA-2Na in D_2O. The resonances at 21.28 and 8.91 ppm should be attributed to the carbon in the 3-aminopropyltriethoxysilane unit.

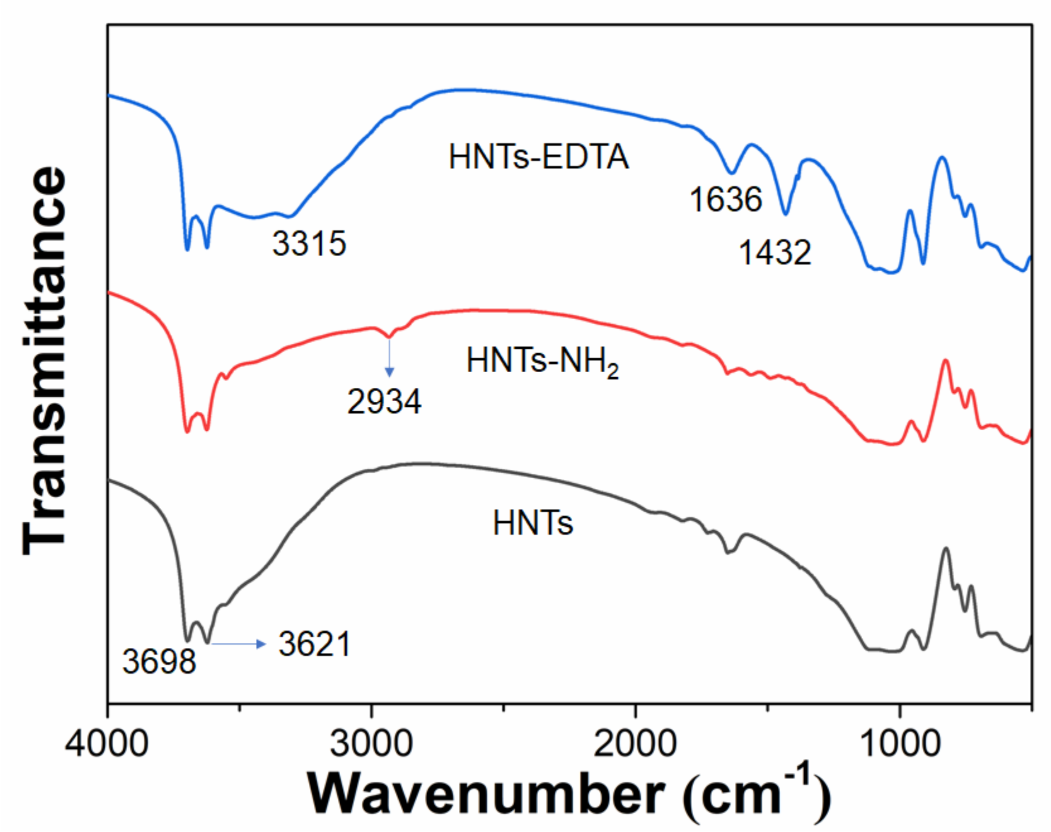

Supplementary Figure 4. Fourier transform infrared spectroscopy results. The broad band from 4000 to 3000 cm^{-1} illustrates the characteristic stretching from HNTs, which should be assigned to the -OH group in aluminosilicate skeletons. The coupling peak at 3698 and 3621 cm^{-1} should be attributed to the -OH groups in different chemical environments: inner surface and the curled layer, which is a typical peculiarity in halloysite nanotubes (HNTs). These characteristic peaks are preserved in the spectra of ethylenediaminetetraacetic acid-derivatized halloysite nanotubes (HNTs-EDTA) and aminated halloysite nanotubes (HNTs-NH_2), implying the basic composition of HNTs did not undergo any serious damage in the modification process. Moreover, the newly emerged peak at 1636 cm^{-1} can be clearly observed, suggesting the presence of -C=O groups in the ethylenediaminetetraacetic acid (EDTA) moieties. The peak at 3315 cm^{-1} should be assigned to the -OH stretching in carboxyl groups.

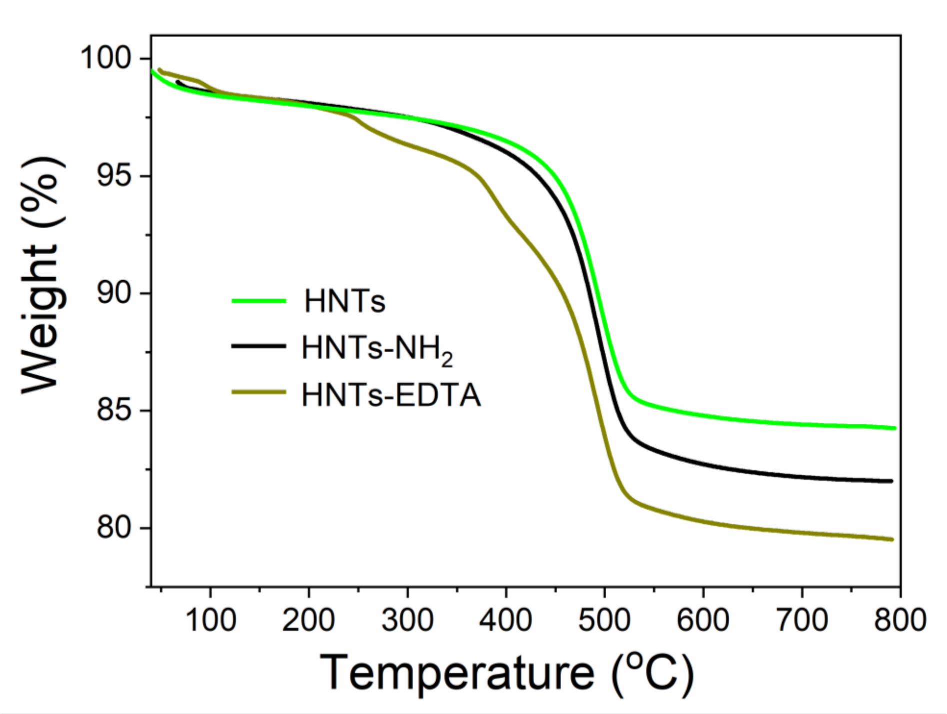

Supplementary Figure 5. Thermal gravimetric results. Thermal gravimetric analysis (TGA) curves of halloysite nanotubes (HNTs), aminated halloysite nanotubes (HNTs-NH_2), and ethylenediaminetetraacetic acid-derivatized halloysite nanotubes (HNTs-EDTA).

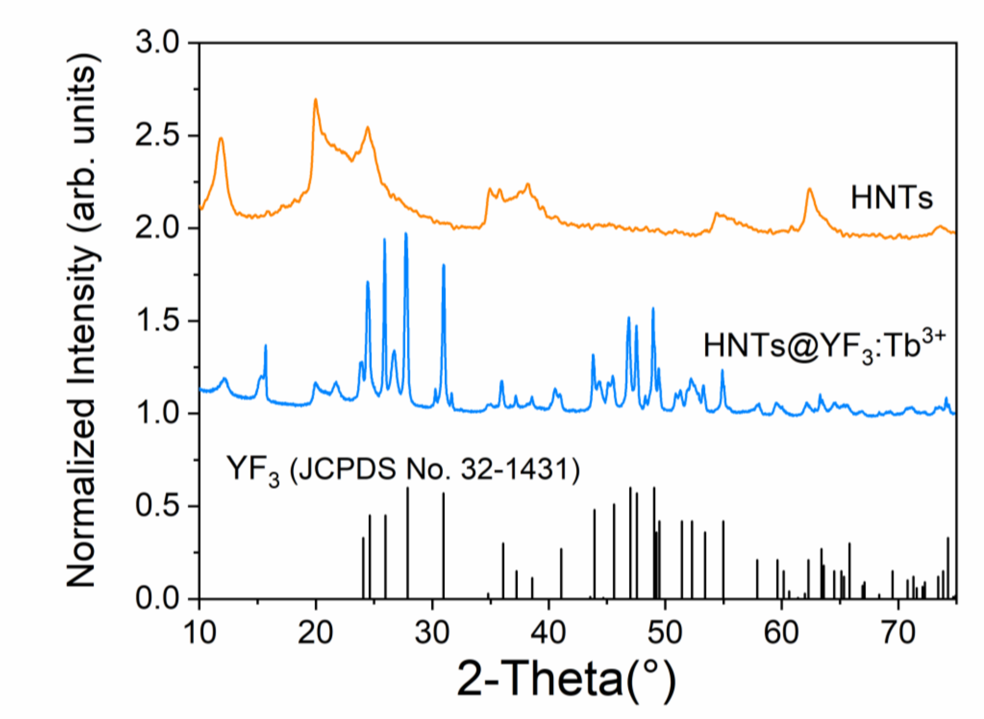

Supplementary Figure 6. X-ray powder diffraction results. Powder X-ray diffraction patterns of halloysite nanotubes (HNTs), halloysite nanotubes-based X-ray-activated visible persistent luminescent emitting phosphors (HNTs@YF_3:Tb^{3+}), and standard of YF_3. JCPDS is abbreviated from Joint Committee on Powder Diffraction Standards.

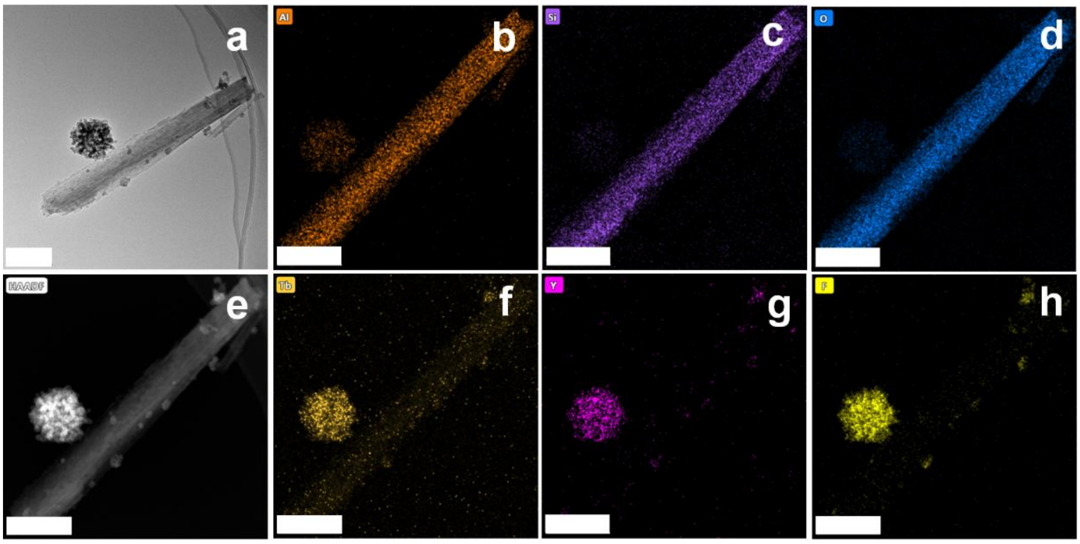

Supplementary Figure 7. Micromorphology characterizations. a Transmission electron microscopy (TEM) image of halloysite nanotubes-based X-ray-activated visible persistent luminescent emitting phosphors (HNTs@YF_3:Tb^{3+}), scale bar: 200 nm. **b** Al distribution image of HNTs@YF_3:Tb^{3+}, scale bar: 200 nm. **c** Si distribution image of HNTs@YF_3:Tb^{3+}, scale bar: 200 nm. **d** O distribution image of HNTs@YF_3:Tb^{3+}, scale bar: 200 nm. **e** High-angle annular dark-field scanning transmission electron microscopy (HAADF-STEM) image, scale bar: 200 nm. **f** Tb distribution image of HNTs@YF_3:Tb^{3+}, scale bar: 200 nm. **g** Y distribution image of HNTs@YF_3:Tb^{3+}, scale bar: 200 nm. **h** F distribution image of HNTs@YF_3:Tb^{3+}, scale bar: 200 nm.

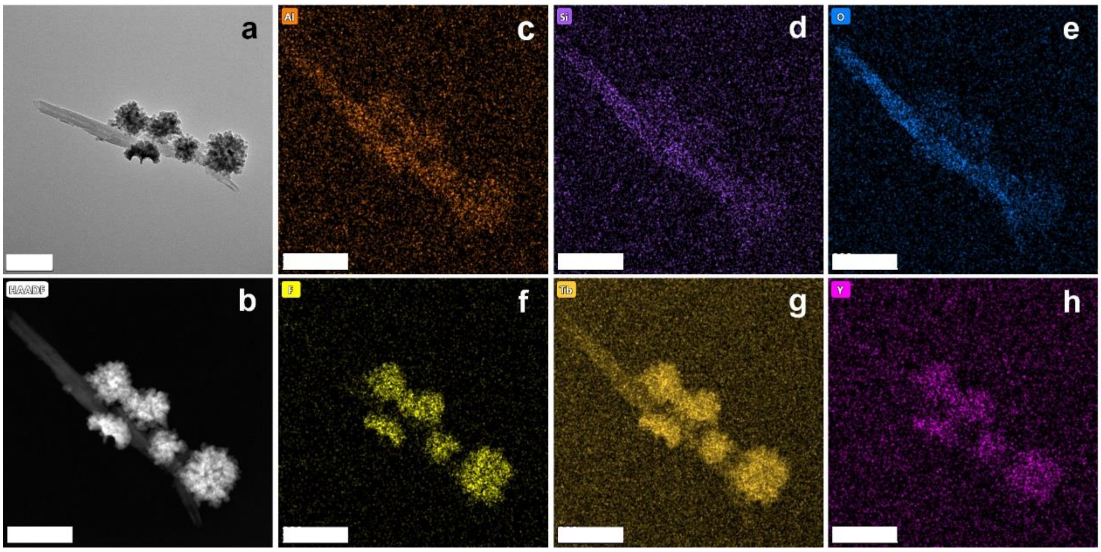

Supplementary Figure 8. Micromorphology characterizations. a Transmission electron microscopy (TEM) image of halloysite nanotubes-based X-ray-activated visible persistent luminescent emitting phosphors (HNTs@YF_3:Tb^{3+}), scale bar: 200 nm. **b** High-angle annular dark-field scanning transmission electron microscopy (HAADF-STEM) image, scale bar: 200 nm. **c** Al distribution image of HNTs@YF_3:Tb^{3+}, scale bar: 200 nm. **d** Si distribution image of HNTs@YF_3:Tb^{3+}, scale bar: 200 nm. **e** O distribution image of HNTs@YF_3:Tb^{3+}, scale bar: 200 nm. **f** F distribution image of HNTs@YF_3:Tb^{3+}, scale bar: 200 nm. **g** Tb distribution image of HNTs@YF_3:Tb^{3+}, scale bar: 200 nm. **h** Y distribution image of HNTs@YF_3:Tb^{3+}, scale bar: 200 nm.

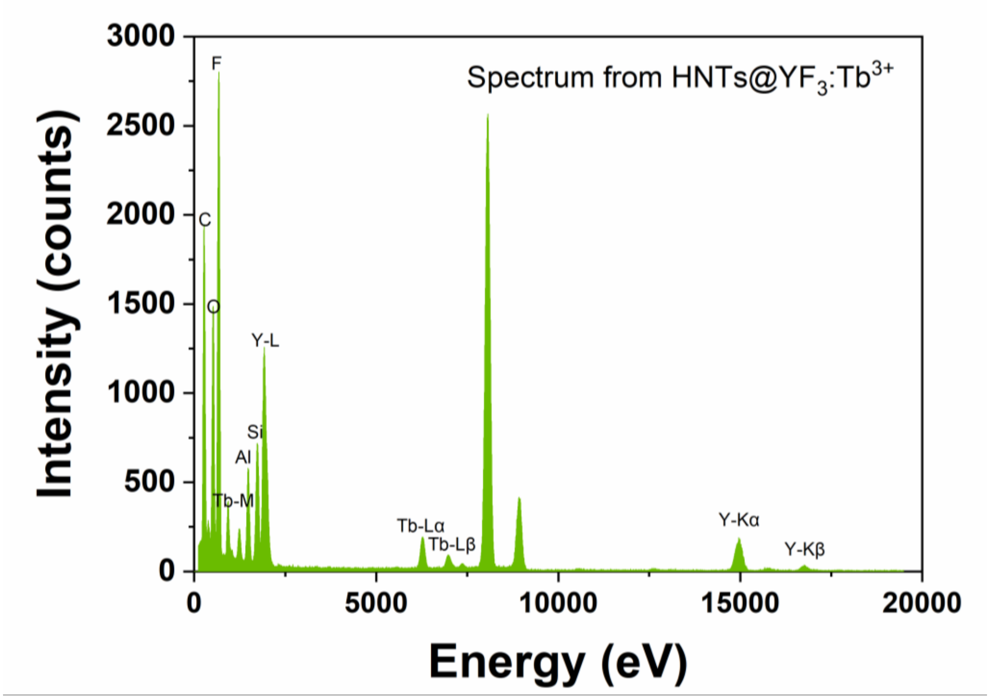

Supplementary Figure 9. Scanning transmission electron microscope-energy dispersive X-ray elemental mapping pattern. Spectrum from halloysite nanotubes-based X-ray-activated visible persistent luminescent emitting phosphors (HNTs@YF_3:Tb^{3+}).

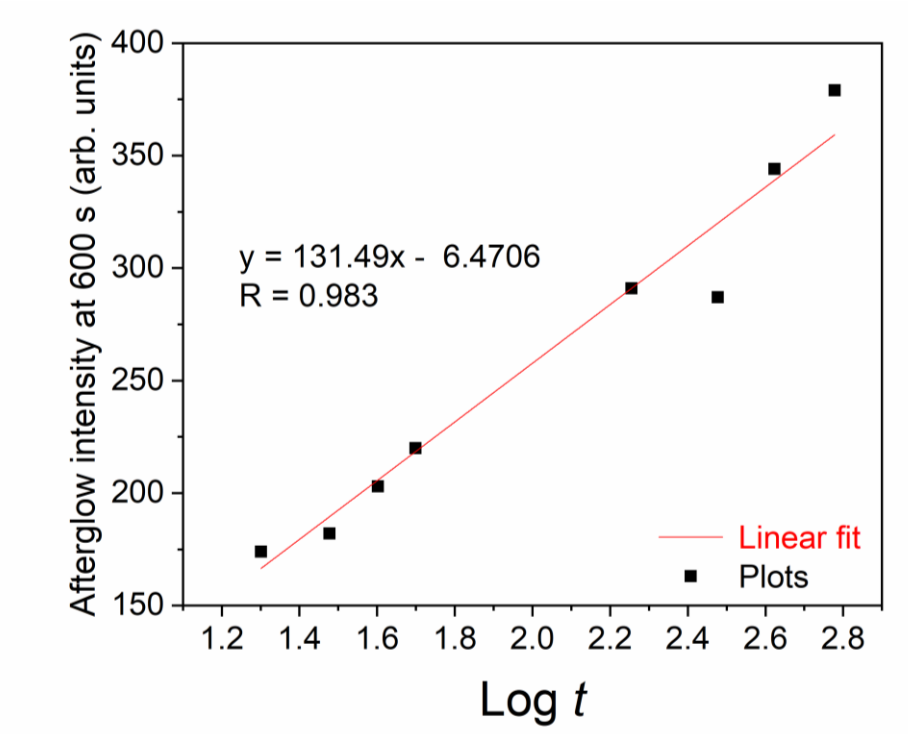

Supplementary Figure 10. Relationship between X-ray irradiation time and intensity of afterglow at 600 s.

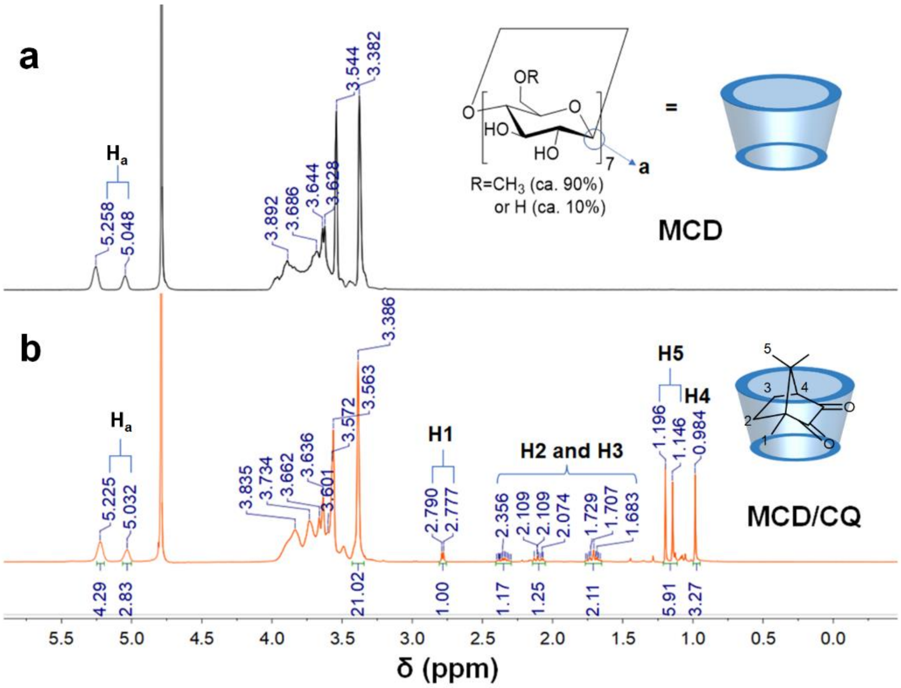

Supplementary Figure 11. Nuclear magnetic resonance spectra of photo initiators. a ^{1}H nuclear magnetic resonance (NMR) spectrum of methyl-β-cyclodextrin (MCD) measured in D_2O. **b** ^{1}H NMR spectrum of the complex of camphorquinone and methyl-β-cyclodextrin (MCD/CQ) measured in D_2O. ^{1}H NMR spectroscopy of MCD/CQ and MCD in D_2O shows the presence of camphorquinone (CQ) with hydrophobic properties. In general, CQ shows a limited water solubility. It is difficult to track the NMR signals by using D_2O. The ^{1}H NMR spectrum of MCD/CQ displays obvious peaks assigned to CQ moiety, implying the water solubility of CQ is improved by the inclusion behavior with MCD. The spectra illustrate the characteristic ^{1}H NMR shifts of the host-guest interaction inclusion compound MCD/CQ compared with unmodified MCD. The integral ratio of H1 (CQ) to H_a (MCD) is calculated as 1:7, suggesting the molar ratio of CQ to MCD should be 1:1. It means the most common association type where a single CQ molecule is included in the one cavity of MCD. The NMR results match well with previously reported studies,[19] suggesting the successful preparation of MCD/CQ.

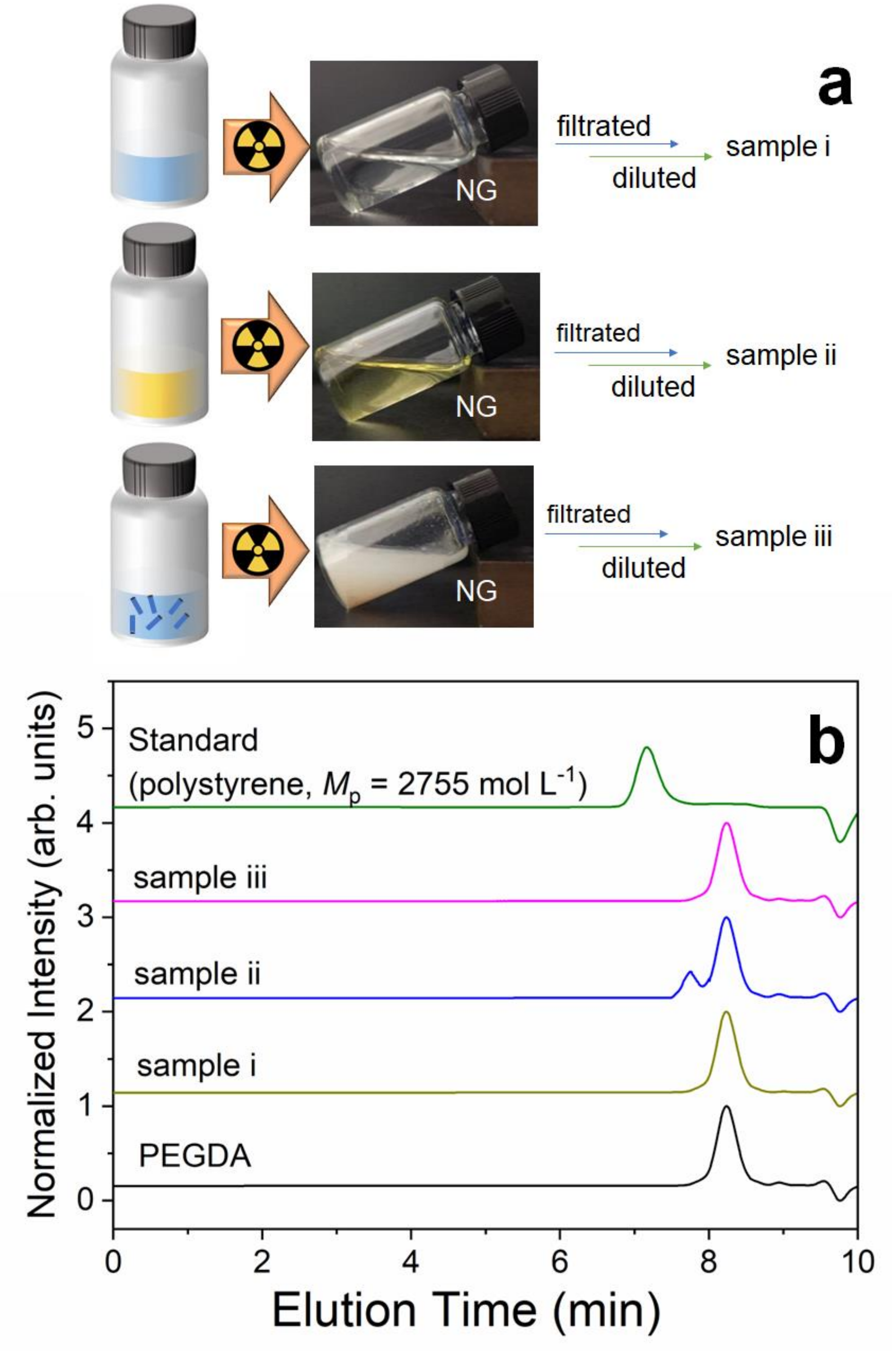

Supplementary Figure 12. Sample preparation and results in parallel experiments. a Sample preparation (i: polyethyleneglycoldiacrylate (PEGDA); ii: PEGDA, complex of camphorquinone and methyl-β-cyclodextrin (MCD/CQ), and triethanolamine; iii: PEGDA and halloysite nanotubes-based X-ray-activated visible persistent luminescent emitting phosphors ($HNTs@YF_3:Tb^{3+}$)). The solutions were exposed to X-ray according to the method depicted in in vitro gelatinization study in main text (NG is abbreviated from not gelatinized). **b** Gel permeation chromatography (GPC) elution curves of PEGDA, samples i, samples ii, samples iii, and standard sample (polystyrene with M_p as 2755 mol L^{-1}).

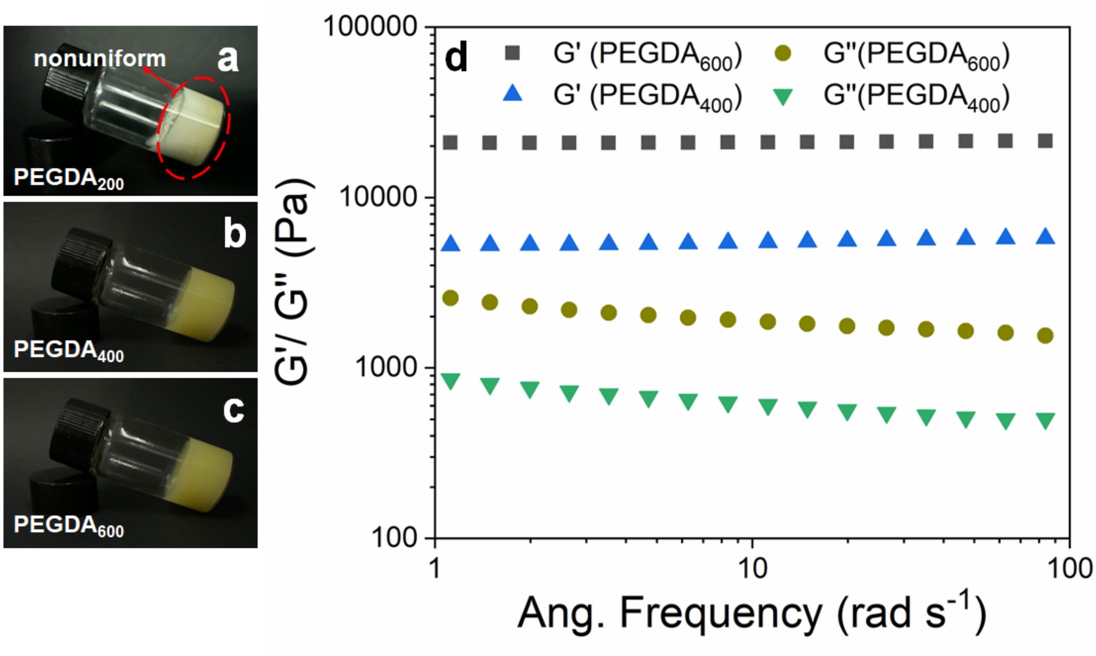

Supplementary Figure 13. Photographs and rheological results of the obtained hydrogels a Photograph of the hydrogel sample by using polyethyleneglycoldiacrylate with molecular weight of 200 g mol^{-1} (PEGDA$_{200}$). The area in red dash lines shows the nonuniform of the formed hydrogel. **b** Photograph of the hydrogel sample by using polyethyleneglycoldiacrylate with molecular weight of 400 g mol^{-1} (PEGDA$_{400}$). **c** Photograph of the hydrogel sample by using polyethyleneglycoldiacrylate with molecular weight of 600 g mol^{-1} (PEGDA$_{600}$). **d** Rheological property of the as-formed hydrogels.

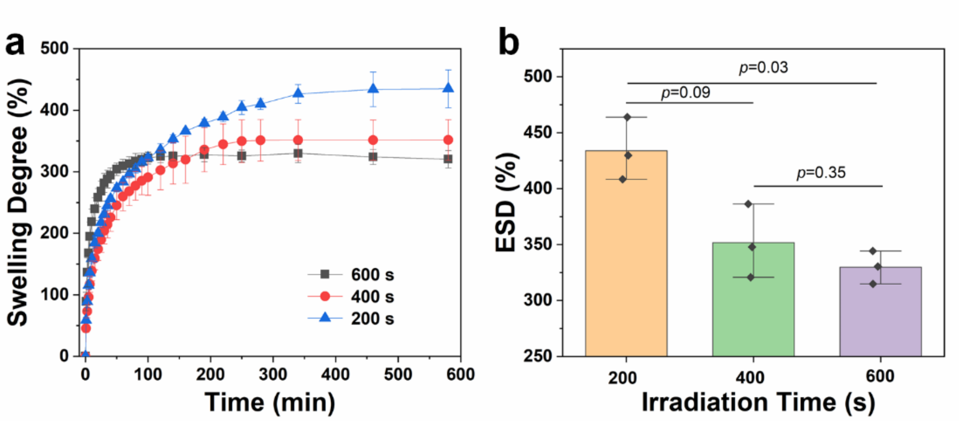

Supplementary Figure 14. Swelling studies of dried hydrogel samples. a Swelling Degree (SD)–time plots of the obtained hydrogel after exposure to X-ray with different times (n=3). **b** Illustration of equilibrium swelling degree (ESD) results (n=3).

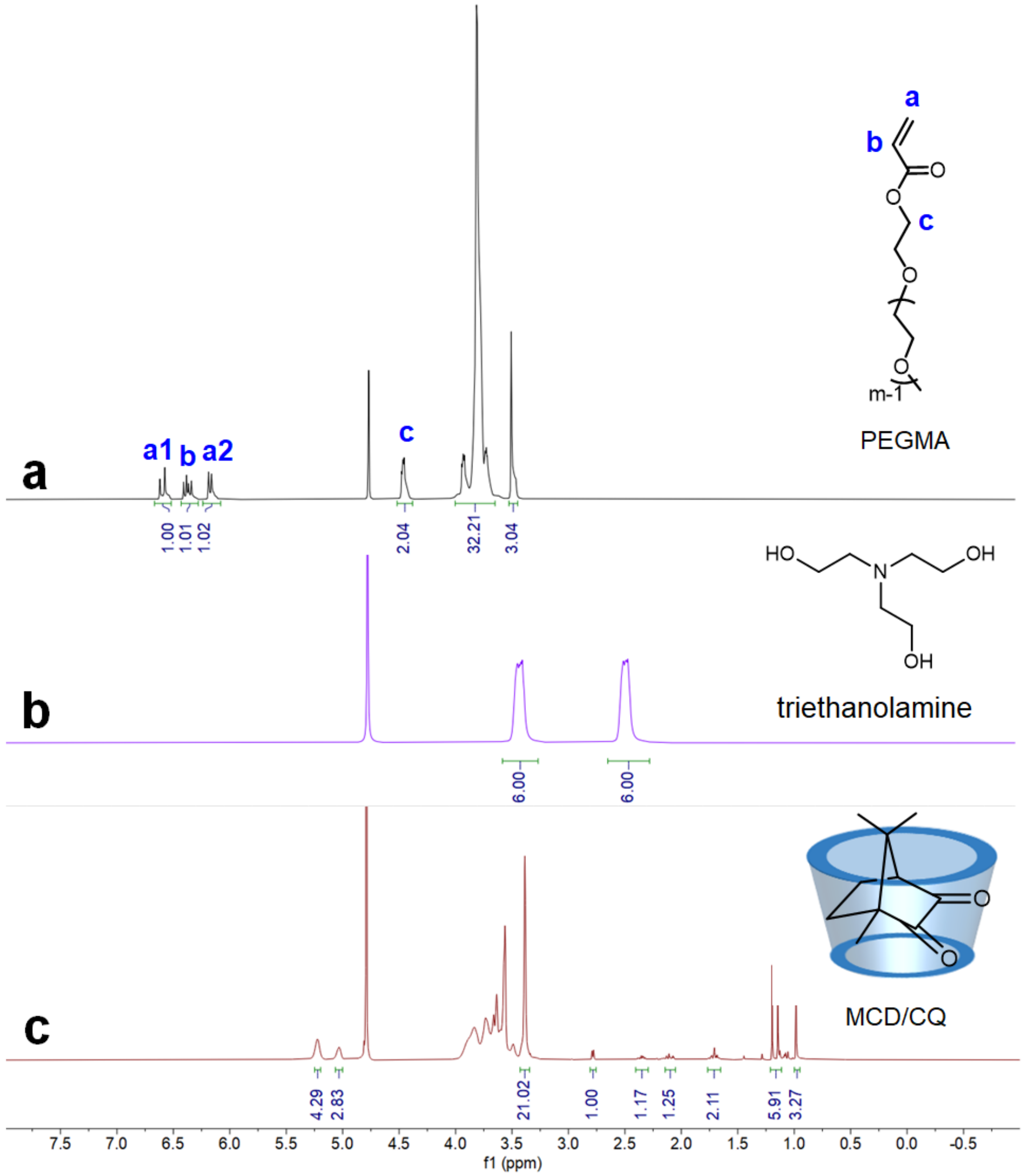

Supplementary Figure 15. Nuclear magnetic resonance results used for calculating conversion. a ^{1}H nuclear magnetic resonance (NMR) spectrum of poly(ethylene glycol) methyl ether acrylate (PEGMA). Peaks labelled as a1 (blue) and a2 (blue) represent the hydrogen a (blue) in the structure of PEGMA. Peaks labelled as b (blue) and c (blue) represent the hydrogen b (blue) and c (blue) in the structure of PEGMA, respectively. **b** ^{1}H NMR spectrum of triethanolamine. **c** ^{1}H NMR spectrum of the complex of camphorquinone and methyl-β-cyclodextrin (MCD/CQ)

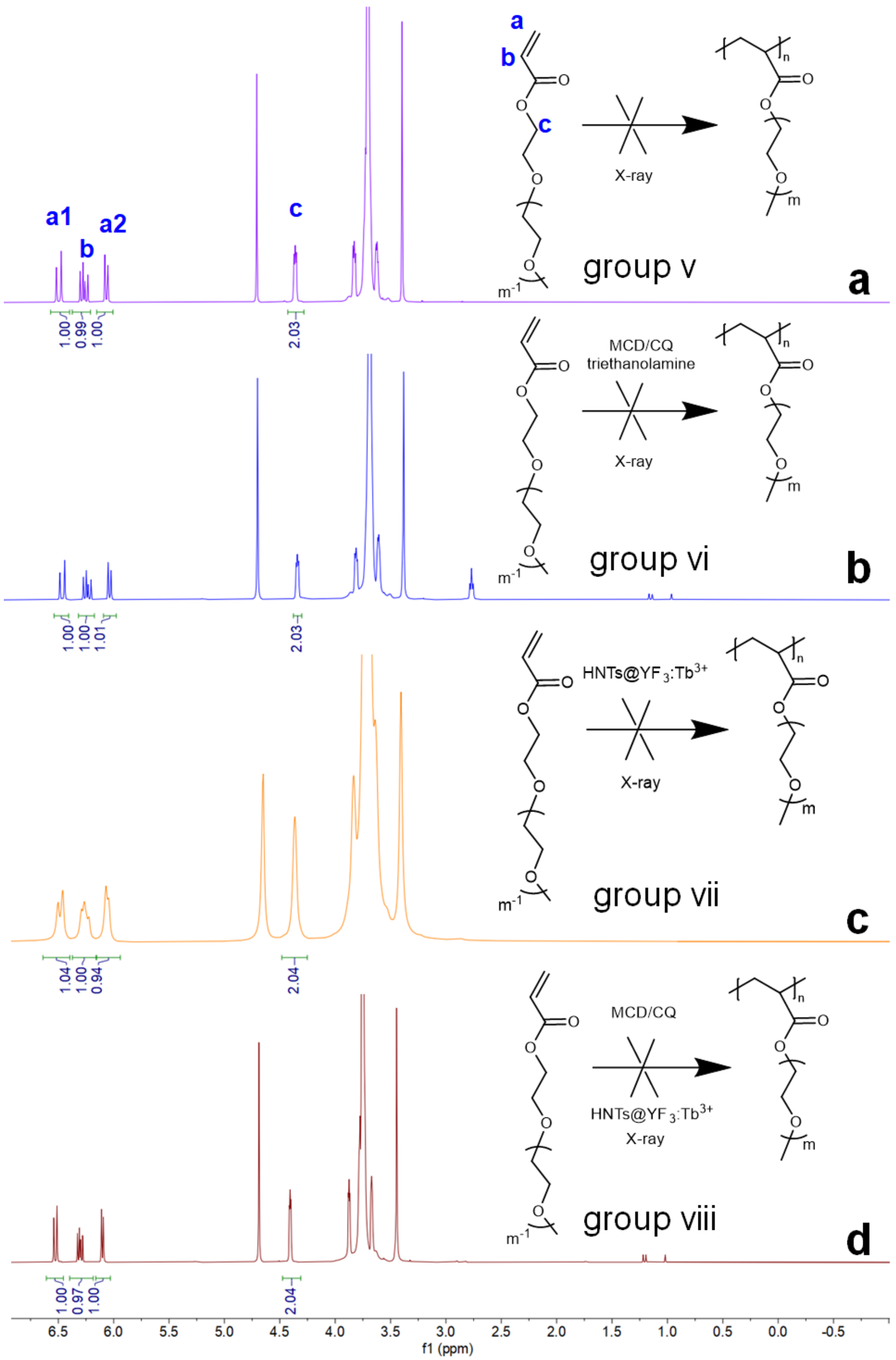

Supplementary Figure 16. Nuclear magnetic resonance results of the samples in parallel experiments. a ^{1}H nuclear magnetic resonance (NMR) spectrum of the sample from group v derived from the solution containing poly(ethylene glycol) methyl ether acrylate (PEGMA). Peaks labelled as a1 (blue) and a2 (blue) represent the hydrogen a (blue) in the structure of

PEGMA. Peaks labelled as b (blue) and c (blue) represent the hydrogen b (blue) and c (blue) in the structure of PEGMA, respectively. **b** ^{1}H NMR spectrum of the sample from group vi derived from the solution containing PEGMA, complex of camphorquinone and methyl-β-cyclodextrin (MCD/CQ), and triethanolamine. **c** ^{1}H NMR spectrum of the sample from group vii derived from the solution containing PEGMA and halloysite nanotubes-based X-ray-activated visible persistent luminescent emitting phosphors (HNTs@YF_3:Tb^{3+}). **d** ^{1}H NMR spectrum of the sample from group viii derived from the solution containing PEGMA, MCD/CQ, and HNTs@YF_3:Tb^{3+}.

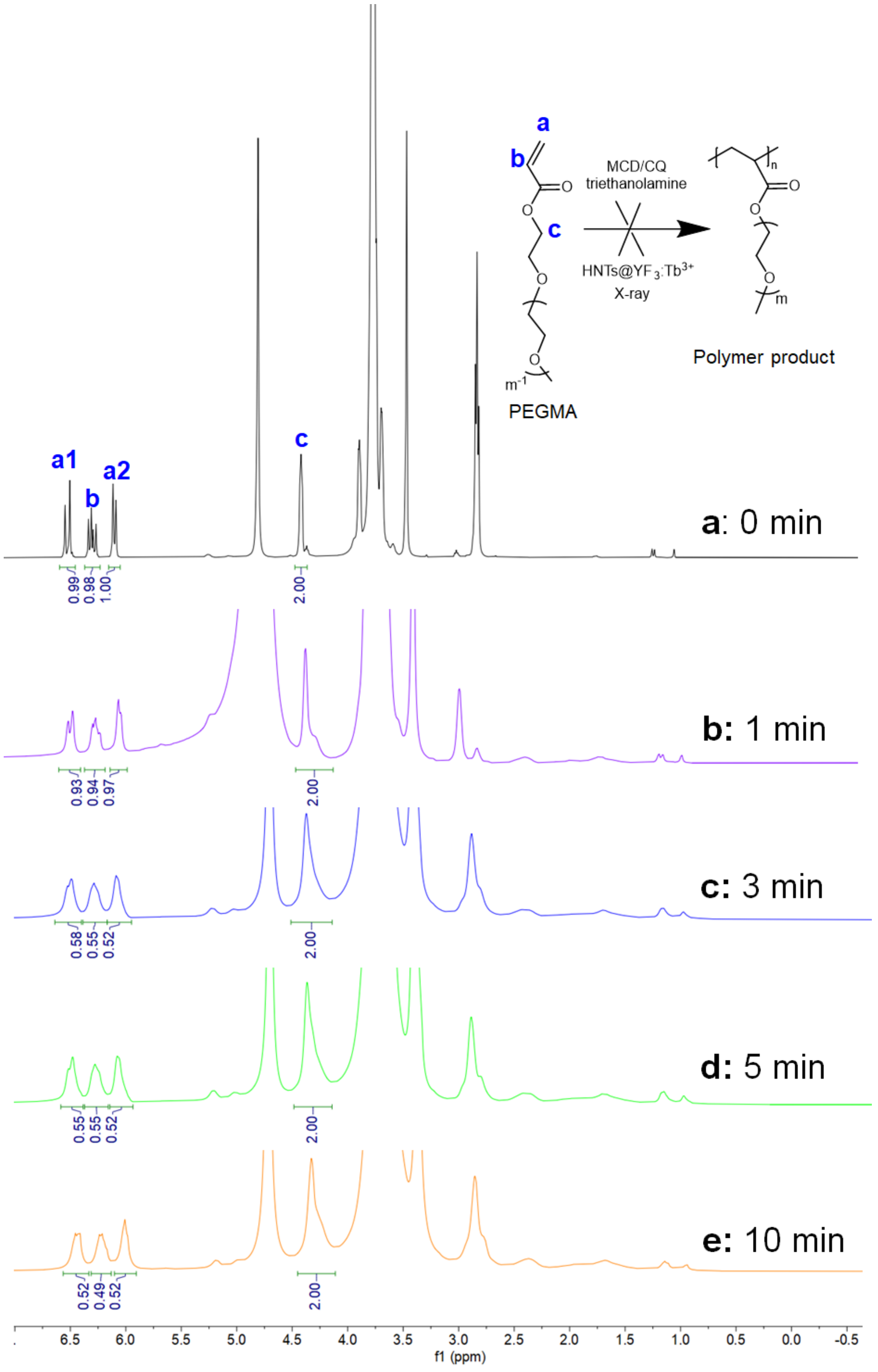

Supplementary Figure 17. Nuclear magnetic resonance results collected at different exposure times. ^{1}H NMR spectra of the D_2O solution containing

halloysite nanotubes-based X-ray-activated visible persistent luminescent emitting phosphors (HNTs@YF_3:Tb^{3+}), poly(ethylene glycol) methyl ether acrylate (PEGMA), triethanolamine, and the complex of camphorquinone and methyl-*β*-cyclodextrin (MCD/CQ) with exposure to X-ray for different times. **a** 0 min. Peaks labelled as a1 (blue) and a2 (blue) represent the hydrogen a in the structure of PEGMA. Peaks labelled as b (blue) and c (blue) represent the hydrogen b (blue) and c (blue) in the structure of PEGMA, respectively. **b** 1 min. **c** 3 min. **d** 5 min. **e** 10min.

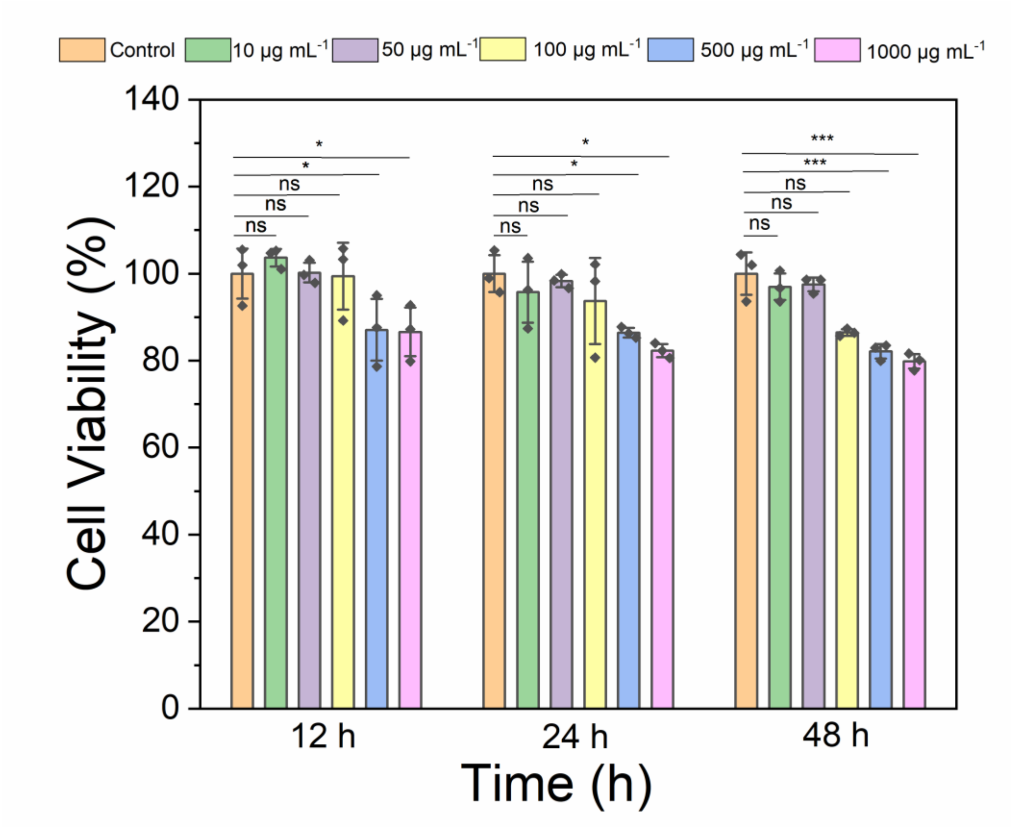

Supplementary Figure 18. Cell viability data in HeLa cells. HeLa cells were seeded in 96-wellplate and then incubated with halloysite nanotubes-based X-ray-activated visible persistent luminescent emitting phosphors (HNTs@YF_3:Tb^{3+}) at 37 °C with the concertation ranging from 10 to 1000 μg mL^{-1} (n=3). Data are presented as mean values +/- standard deviation (SD). The threshold for statistical significance is ***p < 0.001, **p < 0.01, or *p < 0.05. No significant difference is marked as ns.

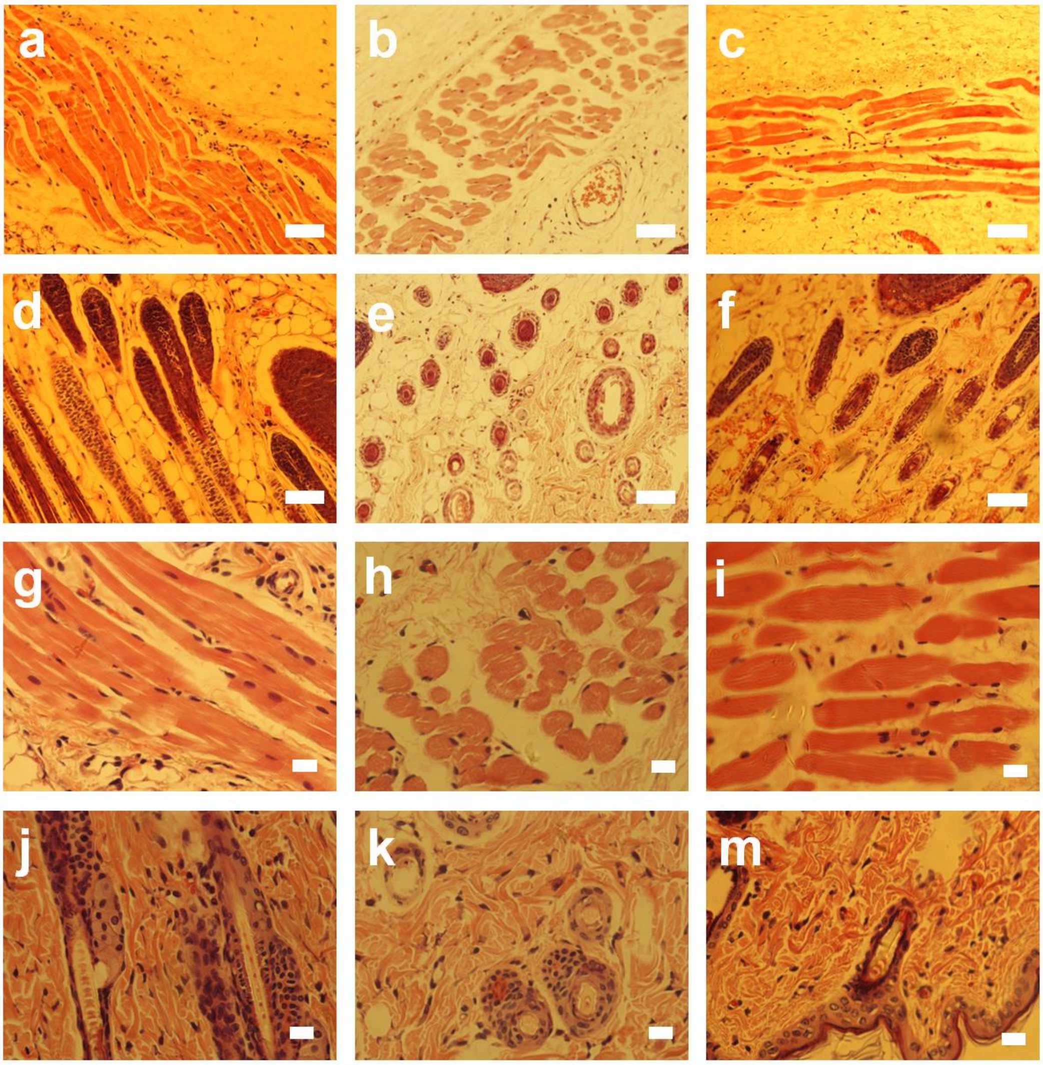

Supplementary Figure 19. Representative photomicrograph of skin tissue sections. a Hematoxylin & eosin (H&E)-stained skin tissue picture from the rat in negative control group. Scale bar: 200 μm. **b** H&E-stained skin tissue picture from the rat in positive control group. Scale bar: 200 μm. **c** H&E-stained skin tissue picture from the rat in test group. Scale bar: 200 μm. **d** H&E-stained skin tissue picture from the rat in negative control group. Scale bar: 200 μm. **e** H&E-stained skin tissue picture from the rat in positive control group. Scale bar: 200 μm. **f** H&E-stained skin tissue picture from the rat in test group. Scale: 200 μm. **g** H&E-stained skin tissue picture from the rat in negative control group. Scale bar: 50 μm. **h** H&E-stained skin tissue picture from the rat in positive control group. Scale bar: 50 μm. **i** H&E-stained skin tissue picture from the rat in test group. Scale bar: 50 μm. **j** H&E-stained skin tissue picture from the rat in negative control group. Scale bar: 50 μm. **k** H&E-stained skin tissue picture from the rat in positive control group. Scale bar: 50 μm. **m** H&E-stained skin tissue picture from the rat in test group. Scale bar: 50 μm.

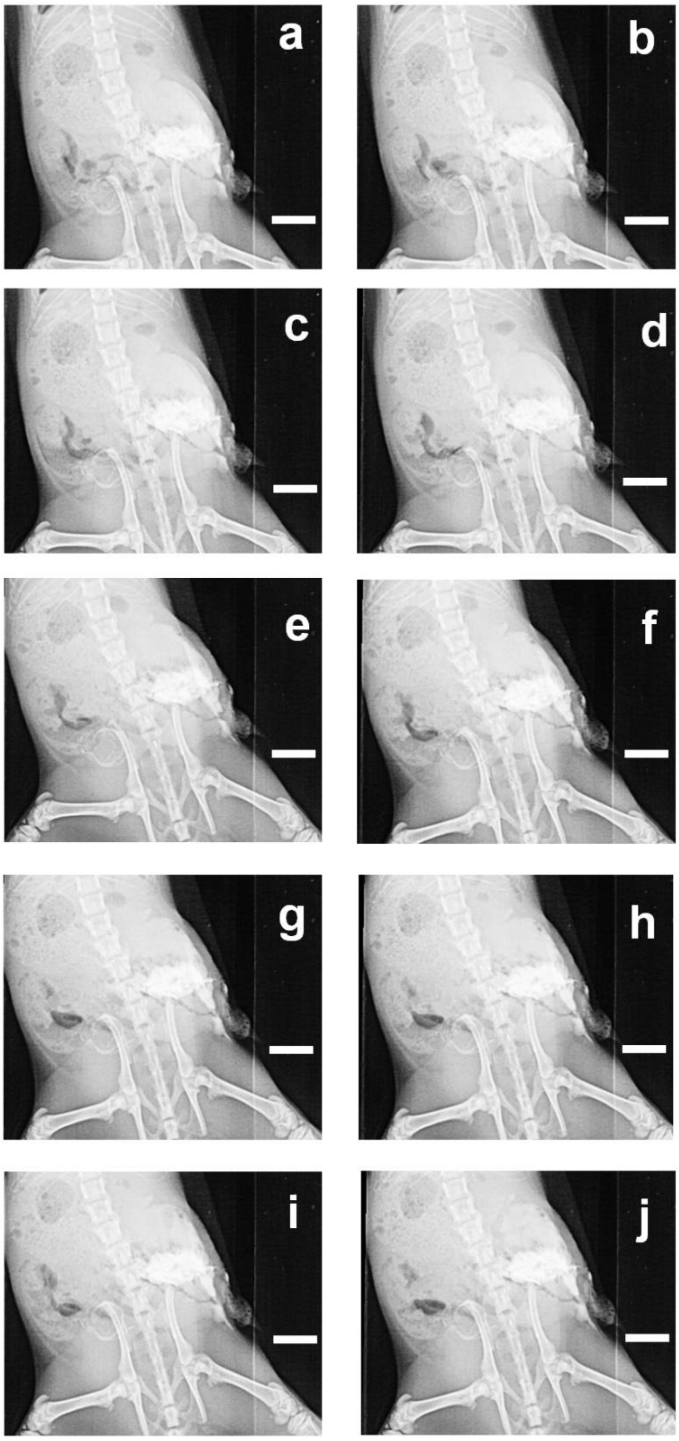

Supplementary Figure 20. X-ray films collected at different times exposure to Sprague Dawley rat. a 1 min. **b** 2 min. **c** 3 min. **d** 4 min. **e** 5 min. **f** 6 min. **g** 7 min. **h** 8 min. **i** 9 min. **j** 10 min. Scale bar: 1 cm.

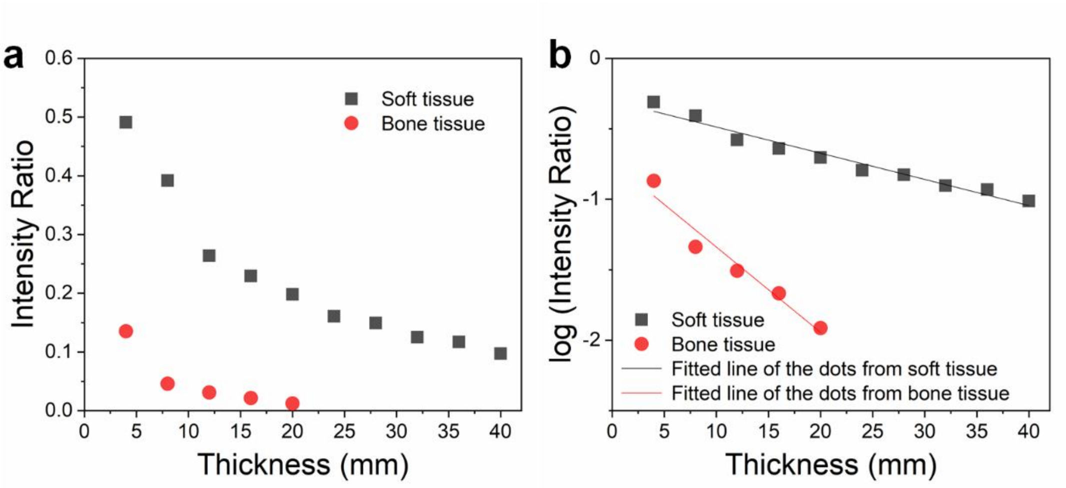

Supplementary Figure 21. Relationship between intensity ratio and tissue thickness. a Plots of intensity ratio vs. thickness in different tissues. **b** Linear relationship between log (intensity ratio) and thickness. Two different kinds of tissues from chicken breast and bovine bones were used in this study. Chicken breast pieces from 4 to 40 mm and bone tissue from 4 to 20 mm were regarded as soft tissue and bone tissue, respectively..

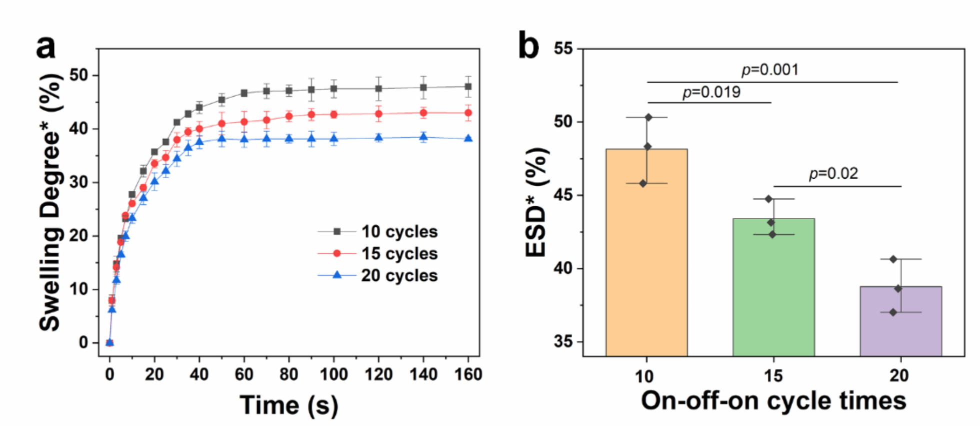

Supplementary Figure 22. Swelling studies of solidified hydrogel samples. a Swelling degree*–time plots of the obtained hydrogel prepared following the on-off-on circulation process with different cycles (10 cycles: 1.5 m Gy; 15 cycles: 2.3 mGy; 20 cycles: 3.0 mGy). **b** The illustration of equilibrium swelling degree measured from the solidified hydrogel sample (ESD*).

河北大学动物实验福利伦理审查同意书

Hebei University Affidavit of Approval of Animal Welfare and Ethical

编号 Approval No.	IACUC- 2021XG008

本《动物实验方案》经过实验动物伦理委员会审核，符合动物保护、动物福利和伦理原则，符合国家实验动物福利伦理的相关规定。方案的相关信息如下：

The animal use protocol listed below has been reviewed and approved by the Animal Ethical and Welfare Committee（AEWC）

课题名称 Protocol Title	基于X射线激发长余辉发光材料构建余辉光诱导水凝胶制备体系<br>Development of afterglow-induced hydrogel based on X-ray-activated long persistent phosphor materials				
申请人 Applicant	张海磊<br>Hailei Zhang	职称/学位 Title/Degree	副教授/博士<br>Associated Professor/PhD	邮箱 Email	zhanghailei1988@126.com
项目负责人 Principle Investigator (PI)	张海磊<br>Hailei Zhang	职称/学位 Title/Degree	副教授/博士<br>Associated Professor/PhD	邮箱 Email	zhanghailei1988@126.com
院系(部门) Department	化学与环境科学学院<br>College of Chemistry and Environmental Science			申请日期 Application date	2021.02.26
动物种系 Species or Strains	家兔、SD鼠<br>Rabbit, Sprague Dawley Rats			动物数量 Quantity	家兔20只，SD鼠30只
计划执行时间 Period of Protocol	2022.01.01-2024.12.31	实验动物使用许可证 Number of Animal use permit		SYXK（冀）2017-002	
审查意见 Results of inspection	☑符合动物福利伦理要求，可以进行实验 Agree<br>☐调整方案后，可以进行实验 Agree after modify				
兽医师 Chief Veterinary Officer	张宏馨		日期 Date	2021.3.11	

河北大学实验动物福利伦理委员会

Animal Welfare and Ethical Committee of Hebei University

主席（或授权人）签章(Chairman's Signature): [illegible]

日期(Date): 2021.3.11

Supplementary Figure 23. Affidavit of approval of animal welfare and ethical

Supplementary References

1 Xu, X. W., Jerca, F. A., Jerca, V. V. & Hoogenboom, R. Covalent poly(2-isopropenyl-2-oxazoline) hydrogels with ultrahigh mechanical strength and toughness through secondary terpyridine metal-coordination crosslinks. *Adv. Funct. Mater.* **29**, 1904886 (2019).

2 Denizli, B. K., Can, H. K., Rzaev, Z. M. O. & Guner, A. Preparation conditions and swelling equilibria of dextran hydrogels prepared by some crosslinking agents. *Polymer* **45**, 6431-6435 (2004).

3 Xu, X., Jerca, F. A., Jerca, V. V. & Hoogenboom, R. Self-healing and moldable poly(2-isopropenyl-2-oxazoline) supramolecular hydrogels based on a transient metal coordination network. *Macromolecules* **53**, 6566-6575 (2020).

4 Ren, P. *et al.* Stiff micelle-crosslinked hyaluronate hydrogels with low swelling for potential cartilage repair. *J. Mater. Chem. B* **7**, 5490-5501 (2019).

5 Zhan, Y., Fu, W., Xing, Y., Ma, X. & Chen, C. Advances in versatile anti-swelling polymer hydrogels. *Mater. Sci. Eng. C* **127**, 112208 (2021).

6 Feng, W. & Wang, Z. Tailoring the swelling-shrinkable behavior of hydrogels for biomedical applications. *Adv. Sci.* **10**, 2303326 (2023).

7 Gao, Y. *et al.* Optimization of a polymer/zeolite-based composite formulation prepared via photopolymerization under near-UV/Visible Light. *ACS Appl. Eng. Mater.* **1**, 3323–3337 (2023).

8 Sirovica, S. *et al.* Origin of micro-scale heterogeneity in polymerisation of photo-activated resin composites. *Nat. Commun.* **11**, 1849 (2020).

9 Leprince, J. G. *et al.* New insight into the “depth of cure” of dimethacrylate-based dental composites. *Dent. Mater.* **28**, 512-520 (2012).

10 Hardy, C. *et al.* Investigating the limits of resin-based luting composite photopolymerization through various thicknesses of indirect restorative materials. *Dent. Mater.* **34**, 1278-1288 (2018).

11 Okulus, Z., Buchwald, T. & Voelkel, A. Characterization of light-cured, dental-resin-based biocomposites. *J. Appl. Polym. Sci.* **132**, 42812 (2015).

12 Weniger, K., Helfmann, J. & Müller, G. Penetration depth of light for argon laser curing of dental composites. *Med. Laser Appl.* **20**, 71-76 (2005).

13 Harlow, J. E., Rueggeberg, F. A., Labrie, D., Sullivan, B. & Price, R. B. Transmission of violet and blue light through conventional (layered) and bulk cured resin-based composites. *J. Dent.* **53**, 44-50 (2016).

14 Haenel, T. *et al.* Effect of the irradiance distribution from light curing units on the local micro-hardness of the surface of dental resins. *Dent. Mater.* **31**, 93-104 (2015).

15 Aravamudhan, K. *et al.* Light-emitting diode curing light irradiance and polymerization of resin-based composite. *J. Am. Dent. Assoc.* **137**, 213-223 (2006).

16 Bakhsh, T. A. *et al.* Effect of light irradiation condition on gap formation under polymeric dental restoration; OCT study. *Z. Med. Phys.* **30**, 194-200 (2020).

17 Hu, P., Xu, H., Pan, Y., Sang, X. & Liu, R. Upconversion particle-assisted NIR polymerization enables microdomain gradient photopolymerization at inter-particulate length scale. *Nat. Commun.* **14**, 3653 (2023).

18 Tseng, S.-J. *et al.* Controlled hydrogel photopolymerization inside live systems by X-

ray irradiation. *Soft Matt.* **8**, 1420-1427 (2012).

19 Zhang, J., Xiao, P., Shi, S. & Nie, J. Preparation and characterization of a water soluble methylated β-cyclodextrin/camphorquinone complex. *Polym. Adv. Technol.* **20**, 723-728 (2009).
