# Supplementary material for: X-ray-activated polymerization expanding the frontiers of deep-tissue hydrogel formation
Source: Nat Commun. 2024 Apr 15;15:3247. doi: 10.1038/s41467-024-47559-z (PMC11018743; doi:10.1038/s41467-024-47559-z)
Supplement: Supplementary file 3 — Reporting Summary [file 41467_2024_47559_MOESM3_ESM.pdf]

nature portfolio

Corresponding author(s): Gang Han

Last updated by author(s): Mar 29, 2024

Reporting Summary

Nature Portfolio wishes to improve the reproducibility of the work that we publish. This form provides structure for consistency and transparency in reporting. For further information on Nature Portfolio policies, see our Editorial Policies and the Editorial Policy Checklist.

Statistics

For all statistical analyses, confirm that the following items are present in the figure legend, table legend, main text, or Methods section.

n/a	Confirmed	
☐	☒	The exact sample size (n) for each experimental group/condition, given as a discrete number and unit of measurement
☒	☐	A statement on whether measurements were taken from distinct samples or whether the same sample was measured repeatedly
☒	☐	The statistical test(s) used AND whether they are one- or two-sided<br>*Only common tests should be described solely by name; describe more complex techniques in the Methods section.*
☒	☐	A description of all covariates tested
☒	☐	A description of any assumptions or corrections, such as tests of normality and adjustment for multiple comparisons
☐	☒	A full description of the statistical parameters including central tendency (e.g. means) or other basic estimates (e.g. regression coefficient) AND variation (e.g. standard deviation) or associated estimates of uncertainty (e.g. confidence intervals)
☒	☐	For null hypothesis testing, the test statistic (e.g. F, t, r) with confidence intervals, effect sizes, degrees of freedom and P value noted<br>*Give P values as exact values whenever suitable.*
☒	☐	For Bayesian analysis, information on the choice of priors and Markov chain Monte Carlo settings
☒	☐	For hierarchical and complex designs, identification of the appropriate level for tests and full reporting of outcomes
☒	☐	Estimates of effect sizes (e.g. Cohen's d, Pearson's r), indicating how they were calculated

Our web collection on statistics for biologists contains articles on many of the points above.

Software and code

Policy information about availability of computer code

Data collection	Origin®(version: b9.5.1.195) is used for graphing and data analysis. Microsoft powerpoint is used for the assembly / layout of figures. TGA was performed on a TA Q50 thermal gravimetric analyzer (TA Instruments, US). X-ray photoelectron spectroscopy (XPS) was carried out on a Thermo Scientific ESCALab 250Xi (Thermo Fisher Scientific, US).Powder X-ray diffraction (PXRD) patterns were recorded on D8 ADVANCE X-ray powder diffractometer system (Bruker Corporation, German).FTIR spectra were recorded on a Nicolet iS10 Fourier transform infrared spectrophotometer (Thermo Fisher Scientific, US). The morphological characterizations, including transmission electron microscope (TEM) observations, energy dispersive X-ray spectroscopy (EDX) maps and high-angle annular dark-field (HAADF) Scanning TEM (STEM) images were performed by using a F200x FEI TalosF200x scanning transmission electron microscope (Thermo Fisher Scientific, US).NMR spectra were obtained on a Bruker 400 M spectrometer (Bruker Corporation, German) or a QOne WNMR-I-400MHz spectrometer (Zhongke-Niujin, China).Gel permeation chromatography (GPC) (Shimazu, Japan) equipped a refractive index detector was used to determine the elution curves of the samples in parallel experiments. AR2000ex-type rotational rheometer (TA, US) was used to investigate the rheological property. Collimalor AL01C II X-Ray Collimator (Type: 5234954; S. N. 7597; Tube current: 100 mA; Tube voltage: 50 kV) was used as the X-ray source and record the X-ray images in the in vivo gelatinization study. H&E and pathology are examined by an Olympus DP 72 microscope camera.
Data analysis	The cell survivals was quantified by using a MTT staining method. The absorbance of each well was measured by the ELX-800 microplate reader (ELISA Reader). Wells without the addition of samples were used as blank control. The cell viability (%) was calculated by the absorbance percentage of test to control. Avantage (version 5.992) is used to analyze the XPS data and and fit the bands in Figure 1. Nano Measurer (version 1.2) is used analyze the lattice in TEM images in Figure 2. MestReNova (version: 14.0.0-23239) is used to perform the NMR analysis. MDI®Jade6 is used to do the XRD analysis.Exact P value was calculated by the one-way ANOVA Tukey's multiple comparisons test.

For manuscripts utilizing custom algorithms or software that are central to the research but not yet described in published literature, software must be made available to editors and reviewers. We strongly encourage code deposition in a community repository (e.g. GitHub). See the Nature Portfolio guidelines for submitting code & software for further information.

Data

Policy information about availability of data

All manuscripts must include a data availability statement. This statement should provide the following information, where applicable:

- Accession codes, unique identifiers, or web links for publicly available datasets
- A description of any restrictions on data availability
- For clinical datasets or third party data, please ensure that the statement adheres to our policy

The data generated in this study are provided in Supplementary Information/Source Data file. The full image dataset is available from the corresponding author upon request

Research involving human participants, their data, or biological material

Policy information about studies with human participants or human data. See also policy information about sex, gender (identity/presentation), and sexual orientation and race, ethnicity and racism.

Reporting on sex and gender	N/A
Reporting on race, ethnicity, or other socially relevant groupings	N/A
Population characteristics	N/A
Recruitment	N/A
Ethics oversight	N/A

Note that full information on the approval of the study protocol must also be provided in the manuscript.

Field-specific reporting

Please select the one below that is the best fit for your research. If you are not sure, read the appropriate sections before making your selection.

☒ Life sciences ☐ Behavioural & social sciences ☐ Ecological, evolutionary & environmental sciences

For a reference copy of the document with all sections, see nature.com/documents/nr-reporting-summary-flat.pdf

Life sciences study design

All studies must disclose on these points even when the disclosure is negative.

Sample size	No statistical method was used to determine the sample size, because the purpose of this study verify the hydrogel formation inside the bodies and it can be recognized by naked eyes.
Data exclusions	no data were excluded
Replication	Experiments were repeated at least three independent experiments with similar results. All experiments were reproduced to reliably support the conclusions stated in the manuscript.
Randomization	The Sprague-Dawley rats were randomly divided into negative control group (n=3), positive control group (n=3) and test group (n=3).
Blinding	Investigators were not blinded to the sample identities during data collection since the formation hydrogel is determined by the X-ray and not not prone to subjective judgment of investigators,

Reporting for specific materials, systems and methods

We require information from authors about some types of materials, experimental systems and methods used in many studies. Here, indicate whether each material, system or method listed is relevant to your study. If you are not sure if a list item applies to your research, read the appropriate section before selecting a response.

Materials & experimental systems

n/a	Involved in the study	
☒	☐	Antibodies
☐	☒	Eukaryotic cell lines
☒	☐	Palaeontology and archaeology
☐	☒	Animals and other organisms
☒	☐	Clinical data
☒	☐	Dual use research of concern
☒	☐	Plants

Methods

n/a	Involved in the study	
☒	☐	ChIP-seq
☒	☐	Flow cytometry
☒	☐	MRI-based neuroimaging

Eukaryotic cell lines

Policy information about cell lines and Sex and Gender in Research

Cell line source(s)	Human cervical cancer cells (HeLa) and mouse fibroblast L929 cells were used in this study, which were purchased from Chinese Academy of Medical Sciences, Peking Union Medical College and the Cell Bank of the Shanghai Chinese Academy of Science, respectively. The cells were maintained in Dulbecco's modified Eagle's medium (DMEM)
Authentication	None of the cell lines have been authenticated.
Mycoplasma contamination	Mycoplasma contamination was tested by using the LookOut PCR detection kit from SigmaAldrich. All cell lines tested negative for mycoplsama contamination.
Commonly misidentified lines (See ICLAC register)	No commonly misidentified cell lines were used.

Animals and other research organisms

Policy information about studies involving animals; ARRIVE guidelines recommended for reporting animal research, and Sex and Gender in Research

Laboratory animals	Sprague-Dawley rats (6-8 weeks old, male) were purchased with protocols approved from the Experimental Animal Center of Hebei Province, Shijiazhuang, China. The protocol has been reviewed and approved by the Animal Ethical and Welfare Committee of Hebei University (Approval No. IACUC-2021XG008, Data: Mar. 11st 2021). Sprague-Dawley rats were fed with regular food and enough water. They were housed in standard plastic cages and place under a certain temperature (25 ± 2oC). The Sprague-Dawley rats were randomly divided into negative control group (n=3), positive control group (n=3) and test group (n=3). All the rats were fasted for 12 h prior to experiments.
Wild animals	The study did not involve wild animals.
Reporting on sex	Lack of sex-base analysis. Because the purpose of this study verify the hydrogel formation inside the bodies. It is independent from sex.
Field-collected samples	The study did not involve samples collected from the field
Ethics oversight	The protocol has been reviewed and approved by the Animal Ethical and Welfare Committee of Hebei University (Approval No. IACUC-2021XG008, Data: Mar. 11st 2021).

Note that full information on the approval of the study protocol must also be provided in the manuscript.
