# Supplementary figures and images for: X-ray-activated polymerization expanding the frontiers of deep-tissue hydrogel formation
Source: Nat Commun. 2024 Apr 15;15:3247. doi: 10.1038/s41467-024-47559-z (PMC11018743; doi:10.1038/s41467-024-47559-z)

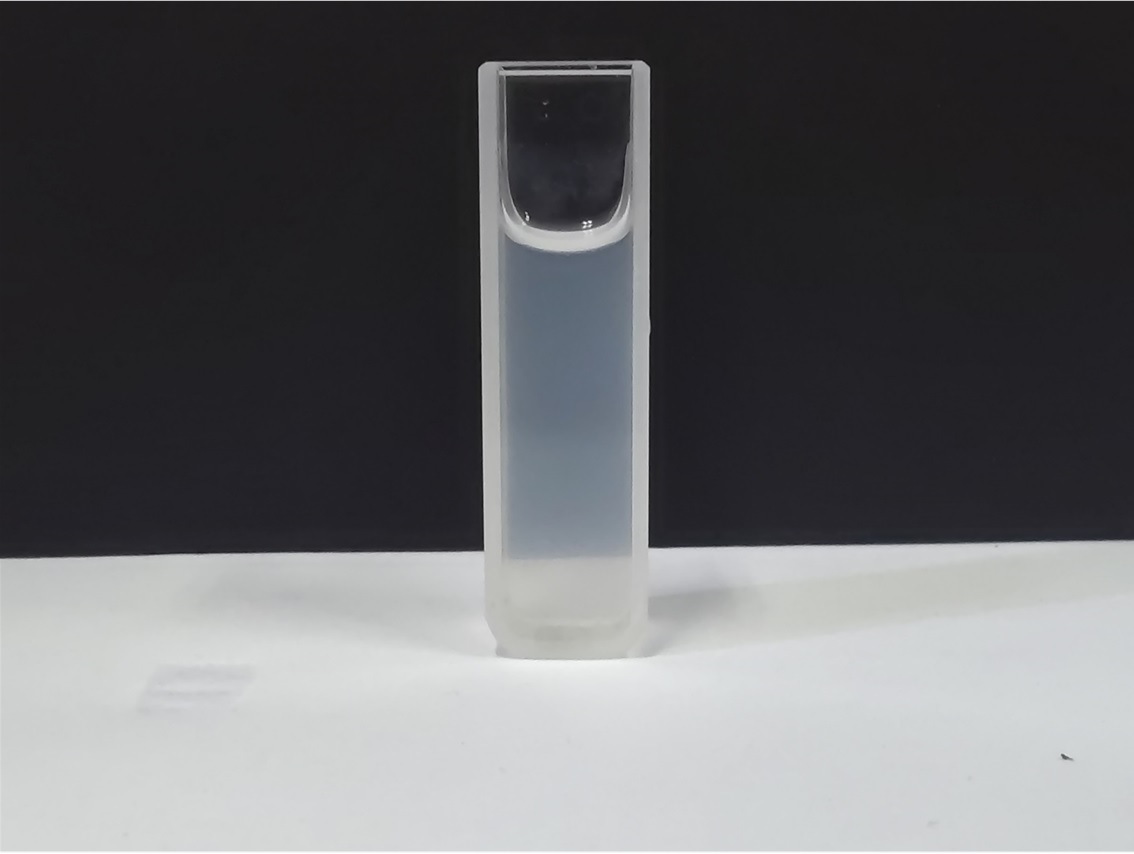

Supplement: Supplementary file 4 — Source Data [file 41467_2024_47559_MOESM4_ESM.zip › Figure 2b.jpg]

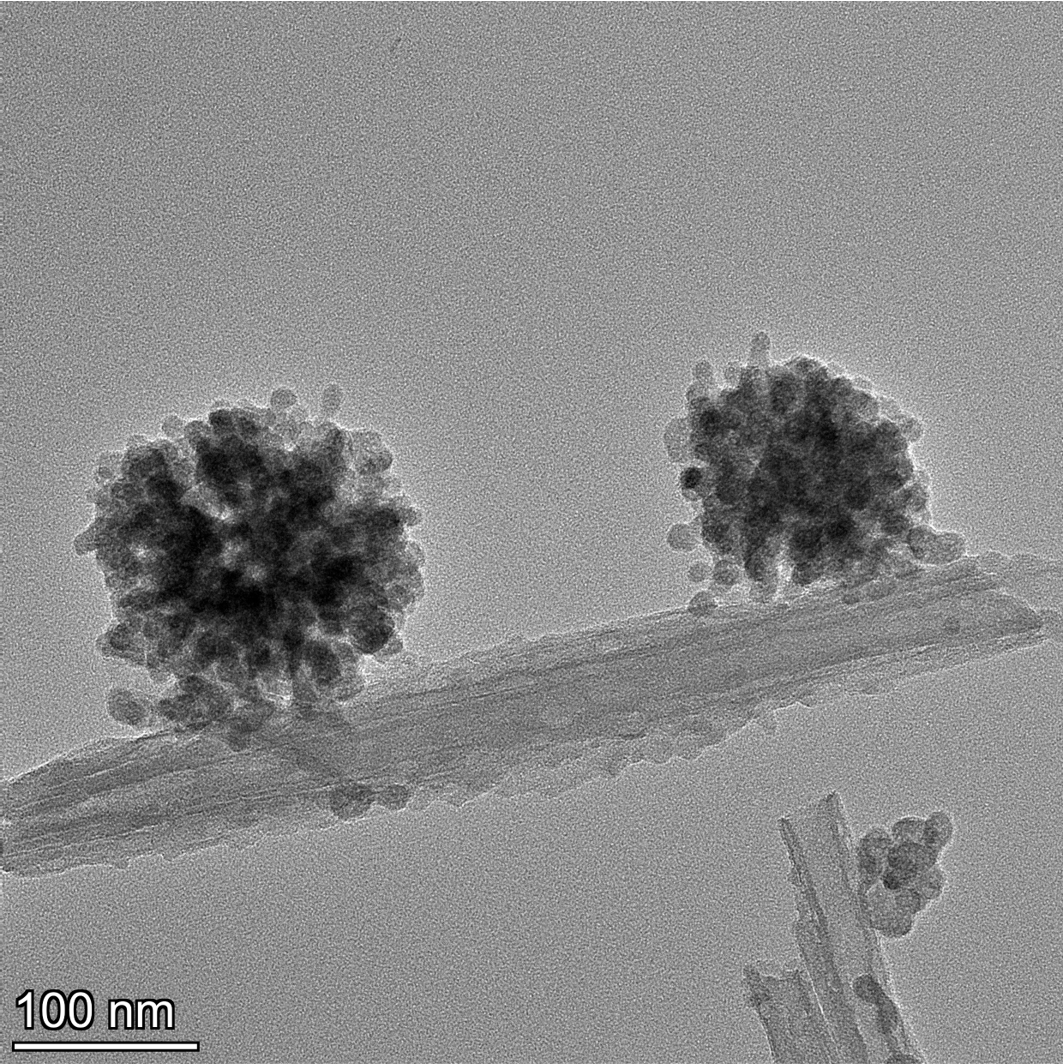

Supplement: Supplementary file 4 — Source Data [file 41467_2024_47559_MOESM4_ESM.zip › Figure 3c.tif]

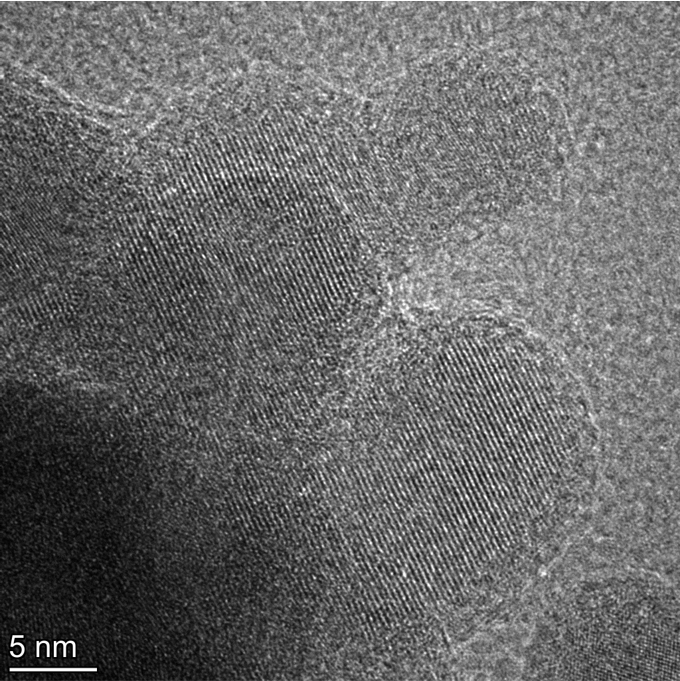

Supplement: Supplementary file 4 — Source Data [file 41467_2024_47559_MOESM4_ESM.zip › Figure 3d.tif]

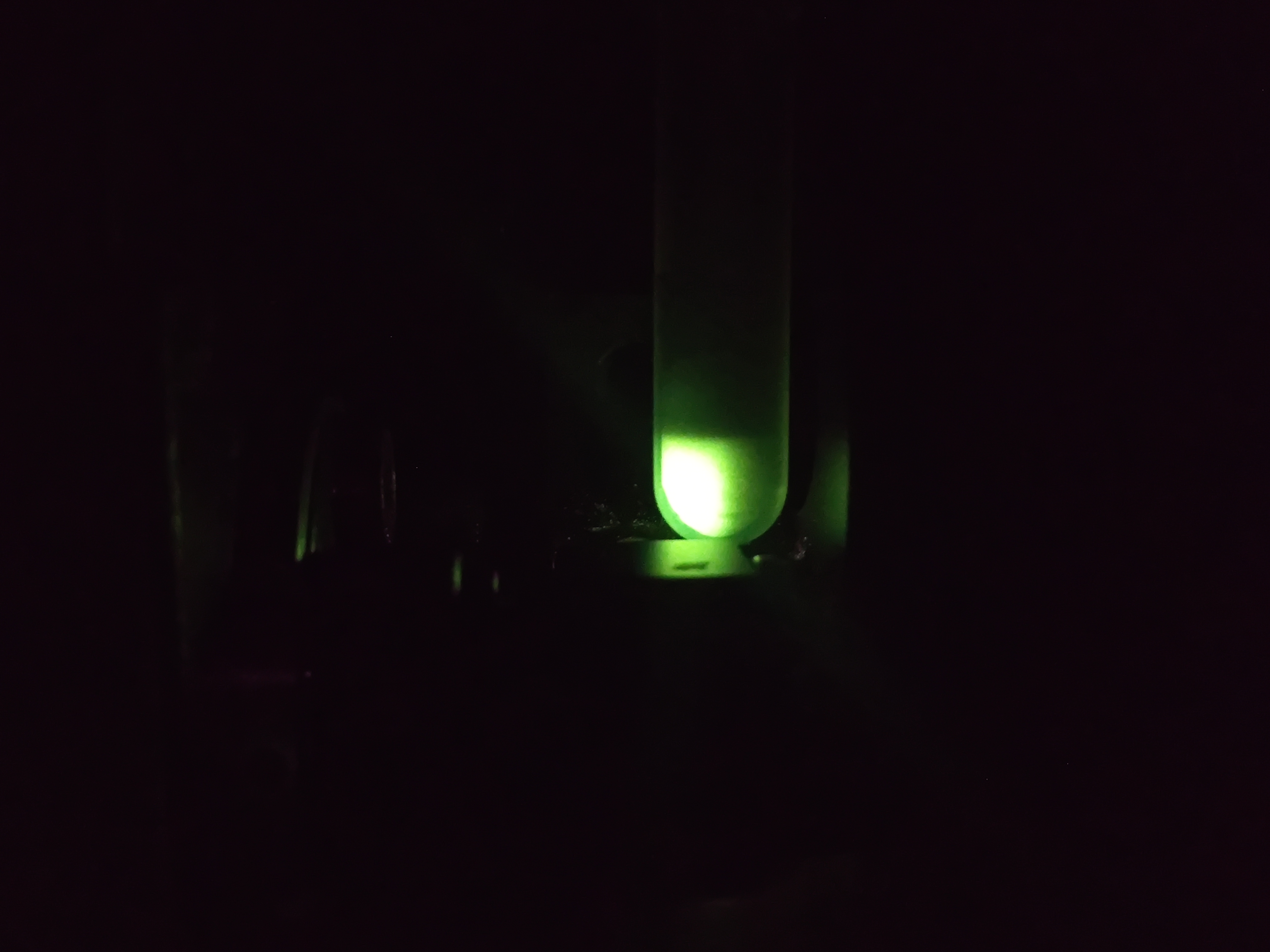

Supplement: Supplementary file 4 — Source Data [file 41467_2024_47559_MOESM4_ESM.zip › Figure 3g.jpg]

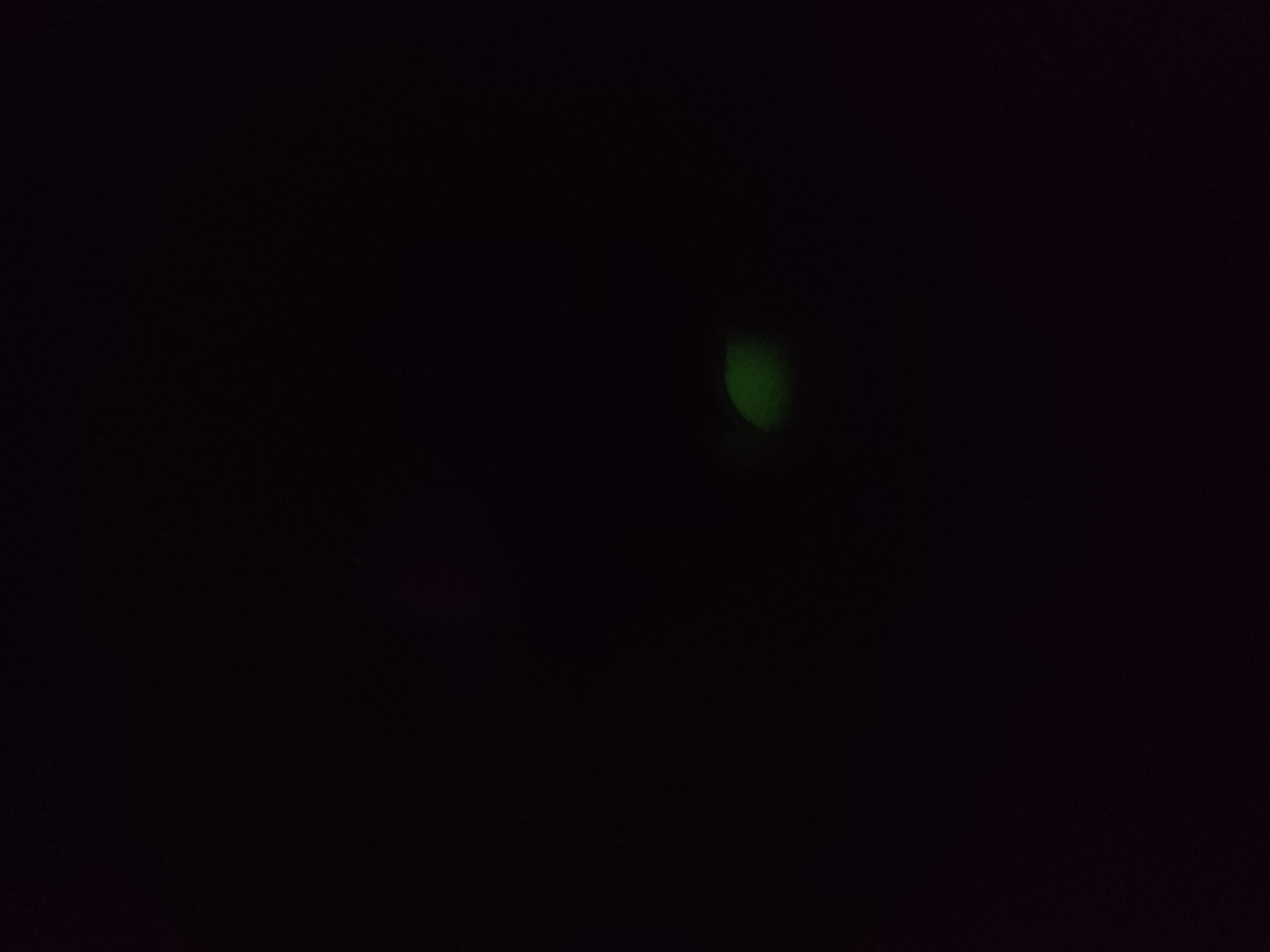

Supplement: Supplementary file 4 — Source Data [file 41467_2024_47559_MOESM4_ESM.zip › Figure 3h.jpg]

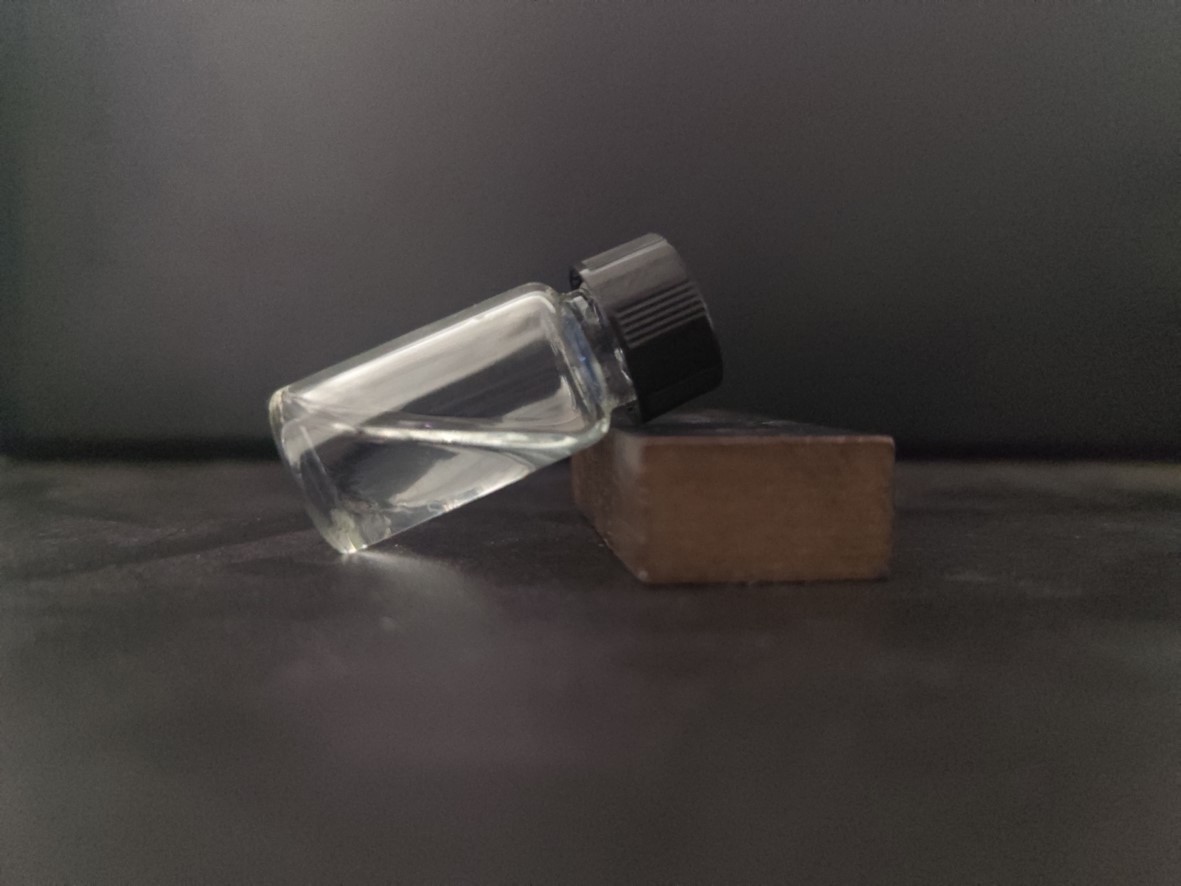

Supplement: Supplementary file 4 — Source Data [file 41467_2024_47559_MOESM4_ESM.zip › Figure 4a-uncropped photogragh.jpg]

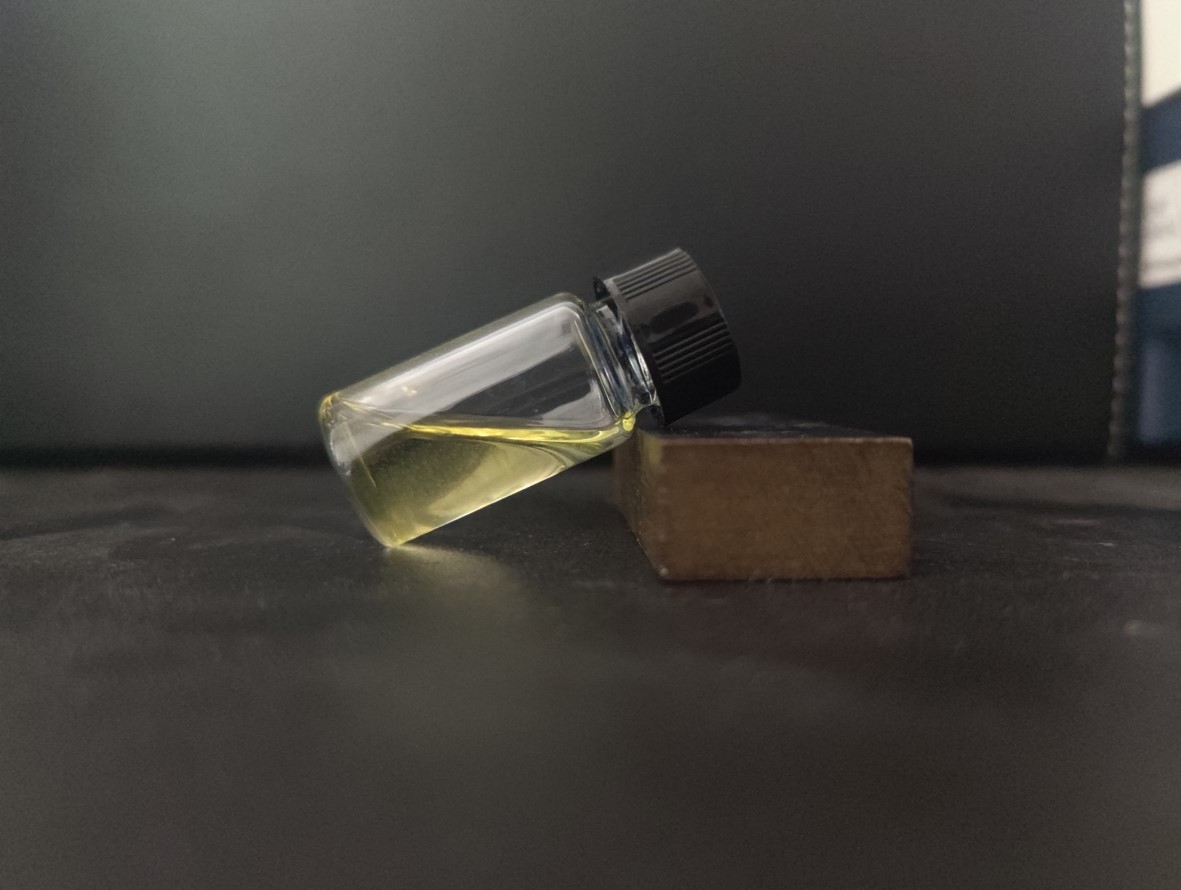

Supplement: Supplementary file 4 — Source Data [file 41467_2024_47559_MOESM4_ESM.zip › Figure 4b-uncropped photogragh.jpg]

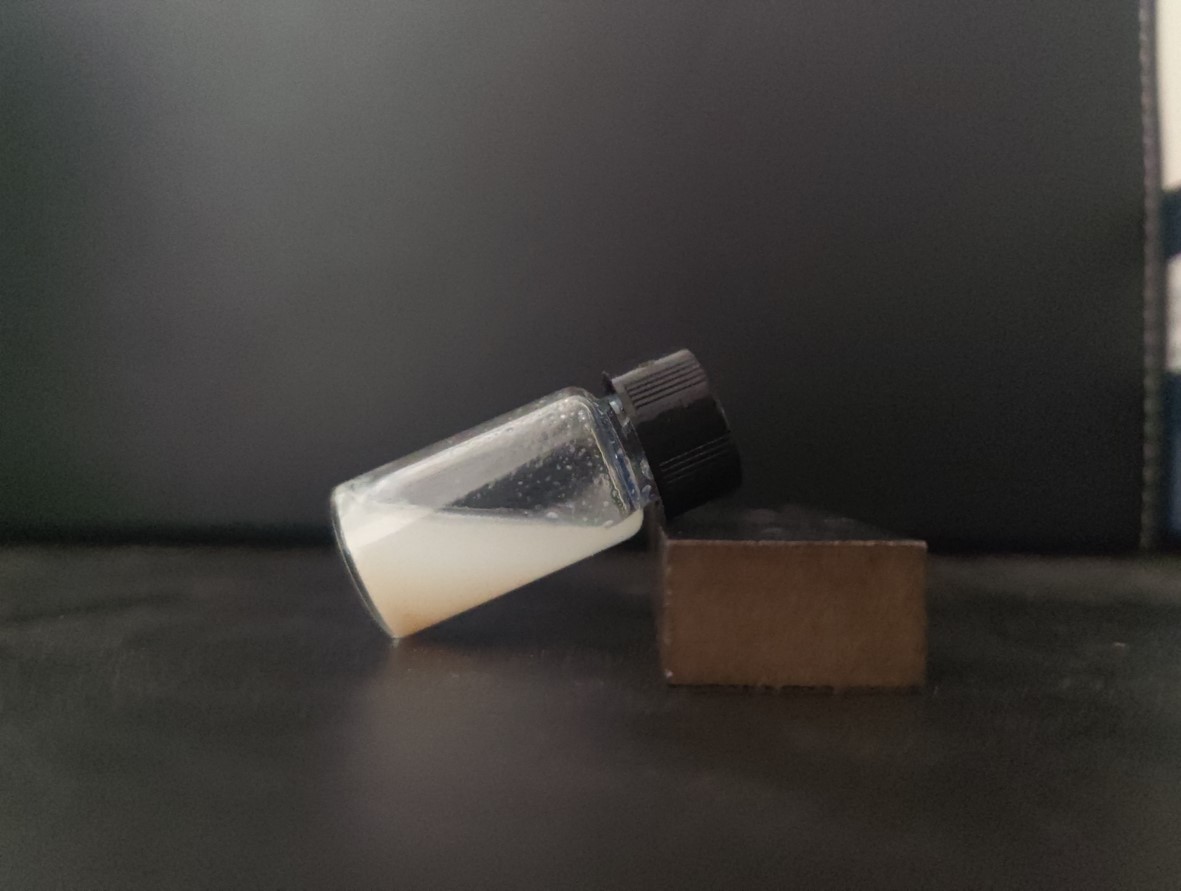

Supplement: Supplementary file 4 — Source Data [file 41467_2024_47559_MOESM4_ESM.zip › Figure 4c-uncropped photogragh.jpg]

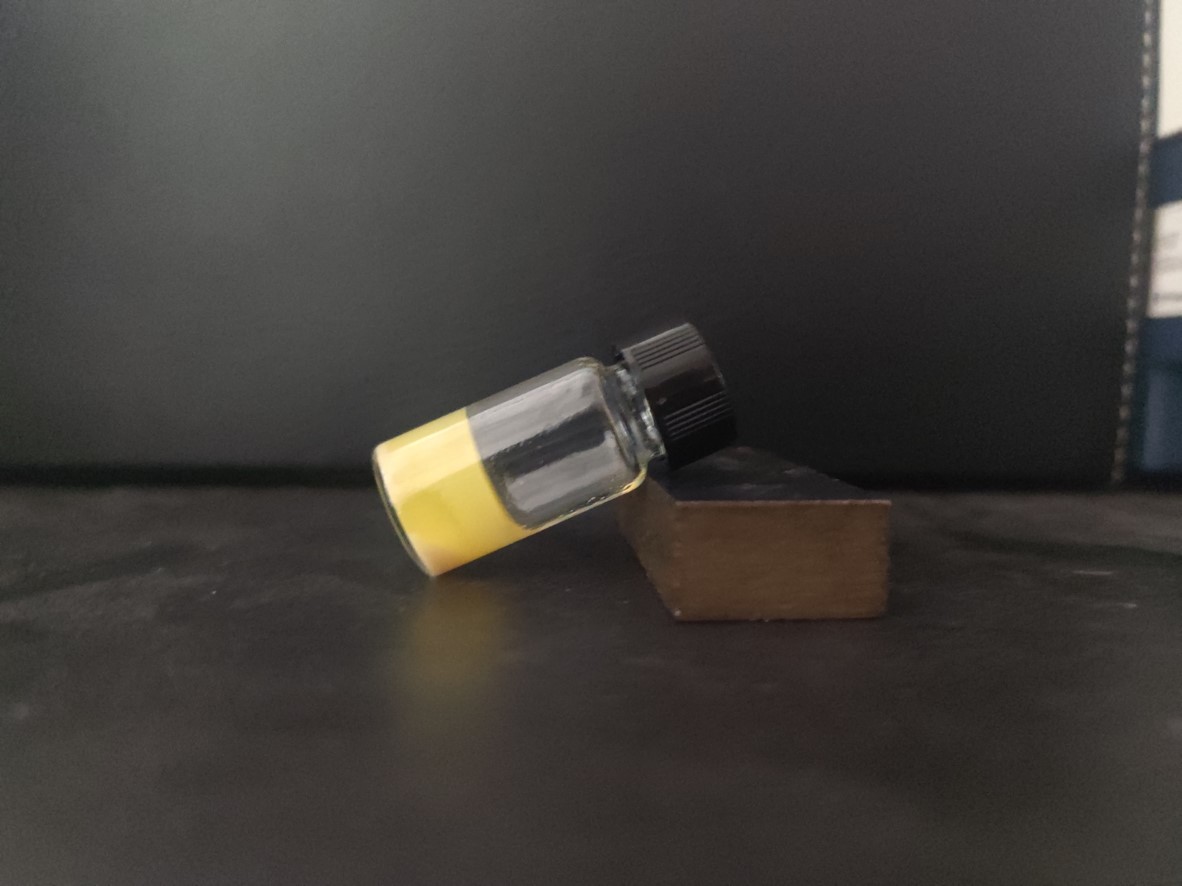

Supplement: Supplementary file 4 — Source Data [file 41467_2024_47559_MOESM4_ESM.zip › Figure 4d-uncropped photogragh.jpg]

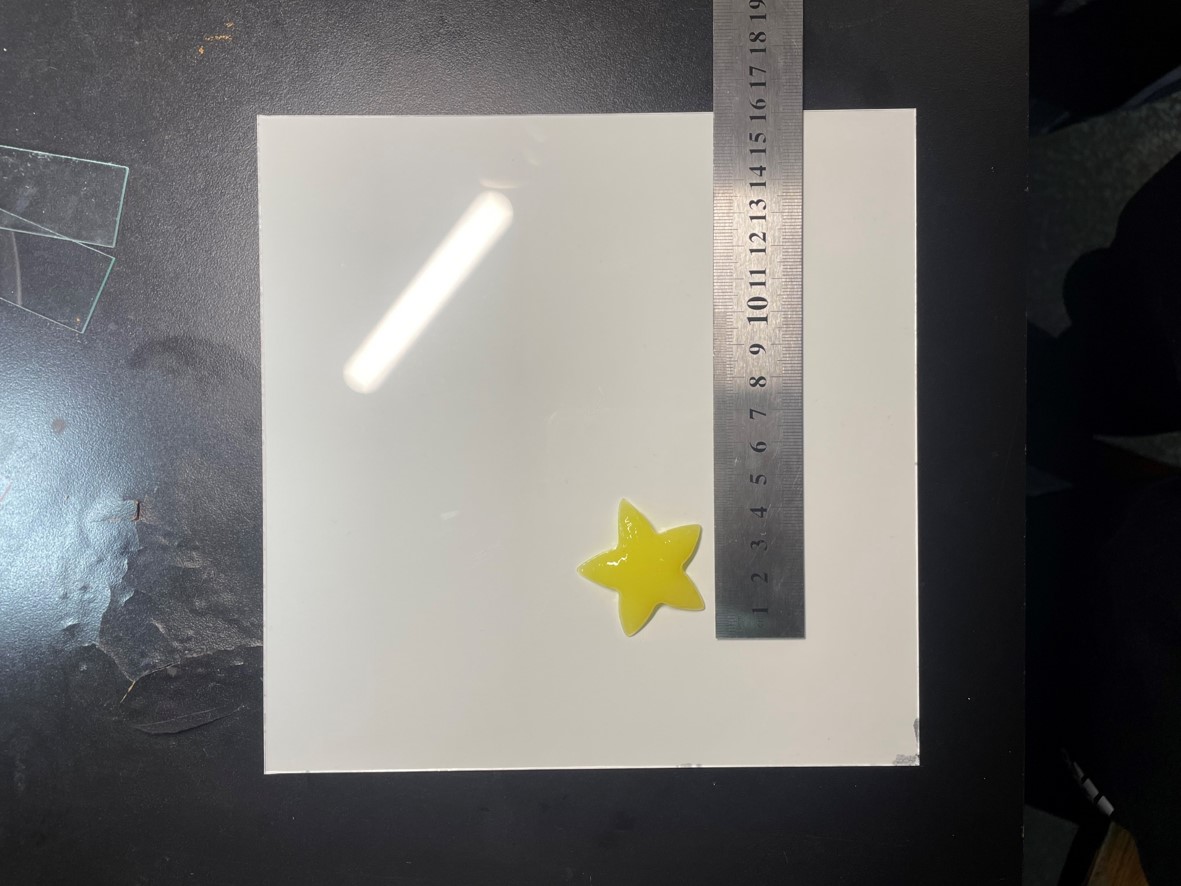

Supplement: Supplementary file 4 — Source Data [file 41467_2024_47559_MOESM4_ESM.zip › Figure 4i-uncropped photogragh.jpg]

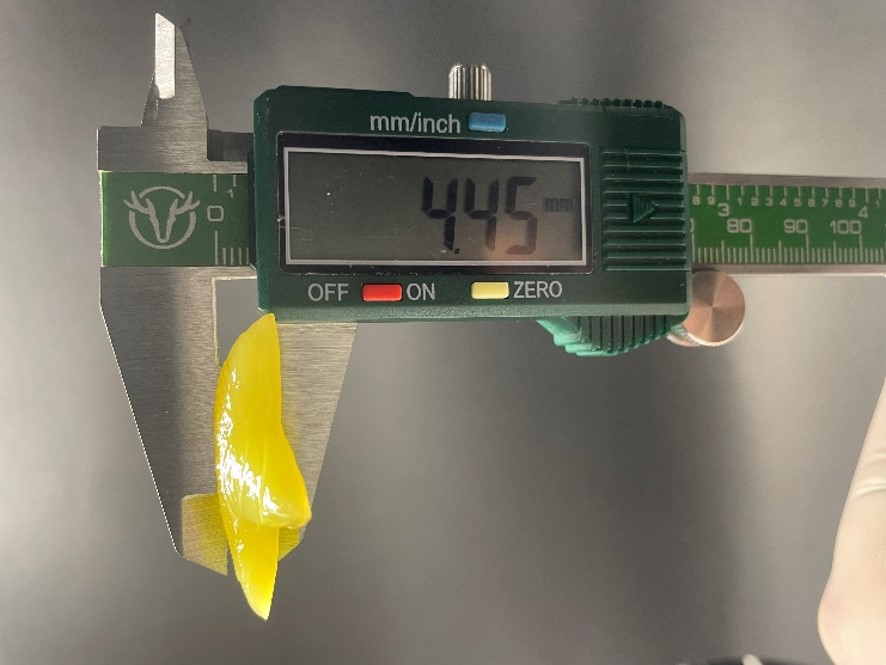

Supplement: Supplementary file 4 — Source Data [file 41467_2024_47559_MOESM4_ESM.zip › Figure 4j-uncropped photogragh.jpg]

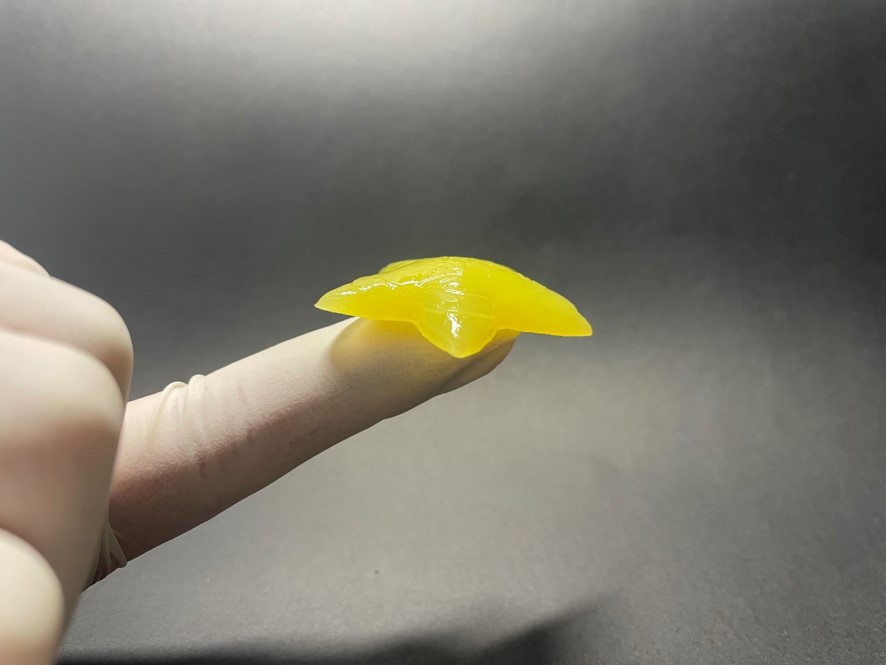

Supplement: Supplementary file 4 — Source Data [file 41467_2024_47559_MOESM4_ESM.zip › Figure 4k-uncropped photogragh.jpg]

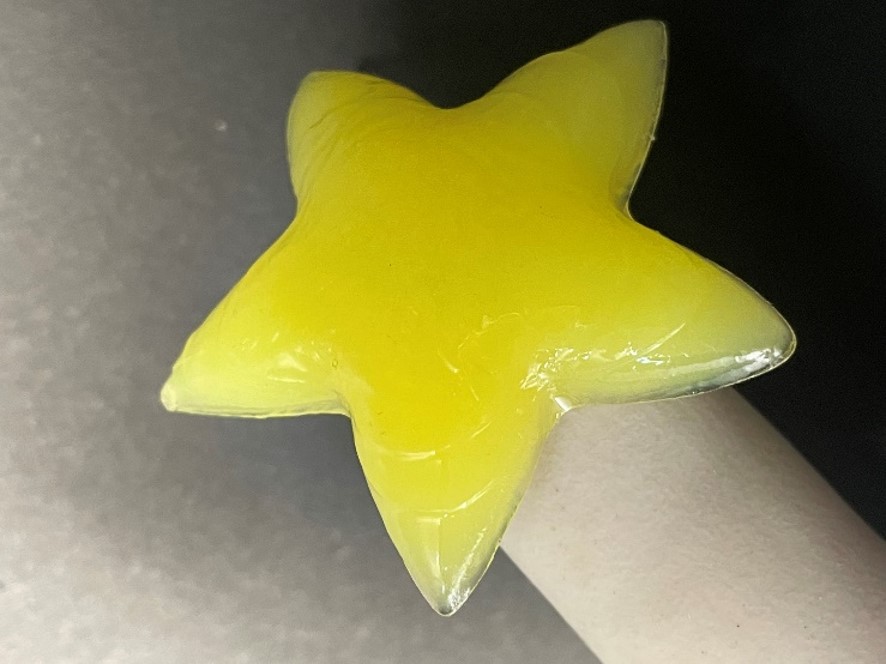

Supplement: Supplementary file 4 — Source Data [file 41467_2024_47559_MOESM4_ESM.zip › Figure 4l-uncropped photogragh.jpg]

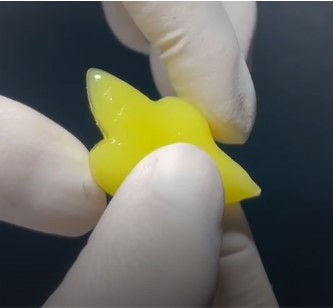

Supplement: Supplementary file 4 — Source Data [file 41467_2024_47559_MOESM4_ESM.zip › Figure 4m-uncropped photogragh.jpg]

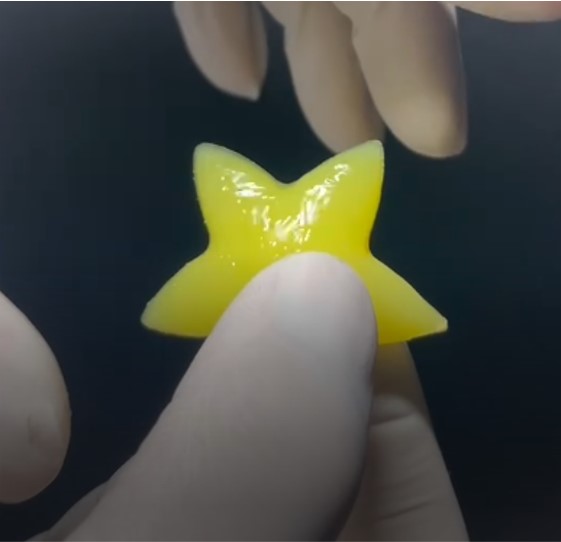

Supplement: Supplementary file 4 — Source Data [file 41467_2024_47559_MOESM4_ESM.zip › Figure 4n-uncropped photogragh.jpg]

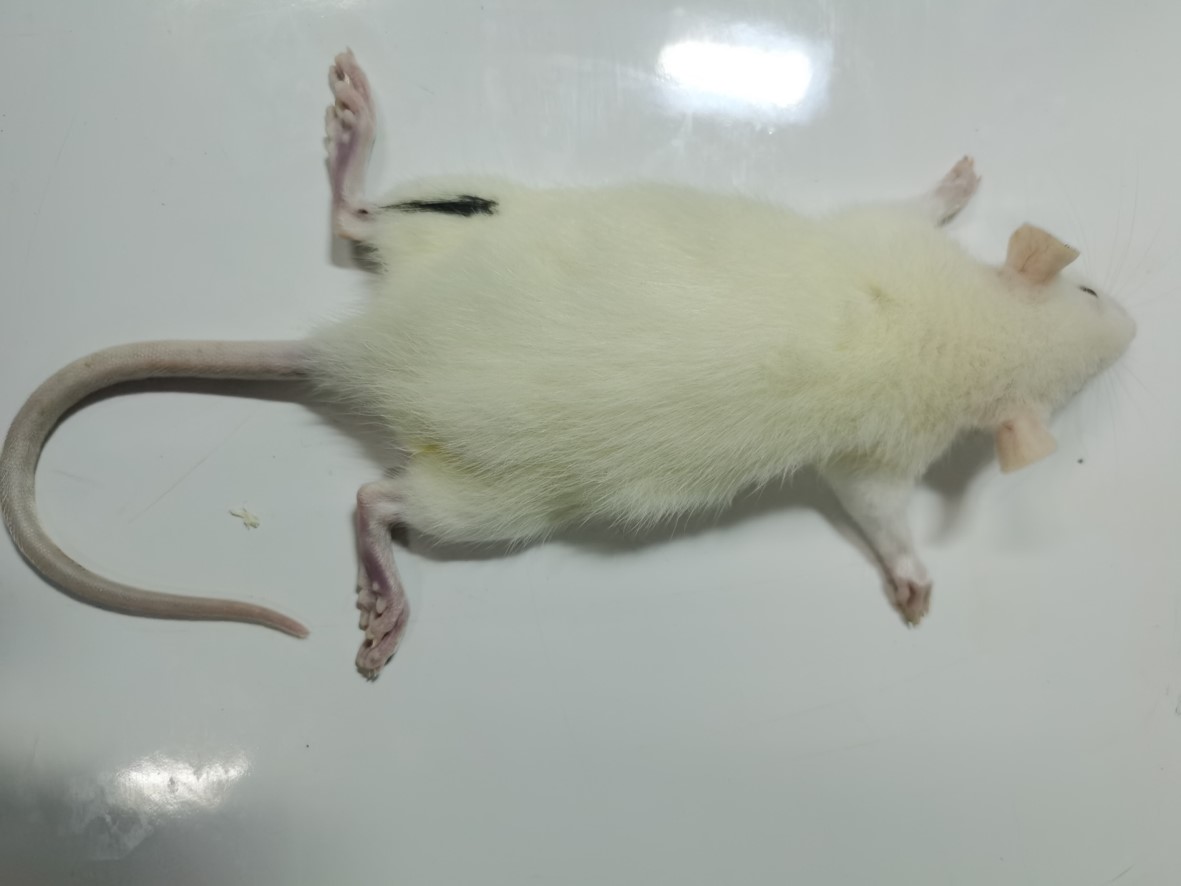

Supplement: Supplementary file 4 — Source Data [file 41467_2024_47559_MOESM4_ESM.zip › Figure 5c-uncropped photogragh.jpg]

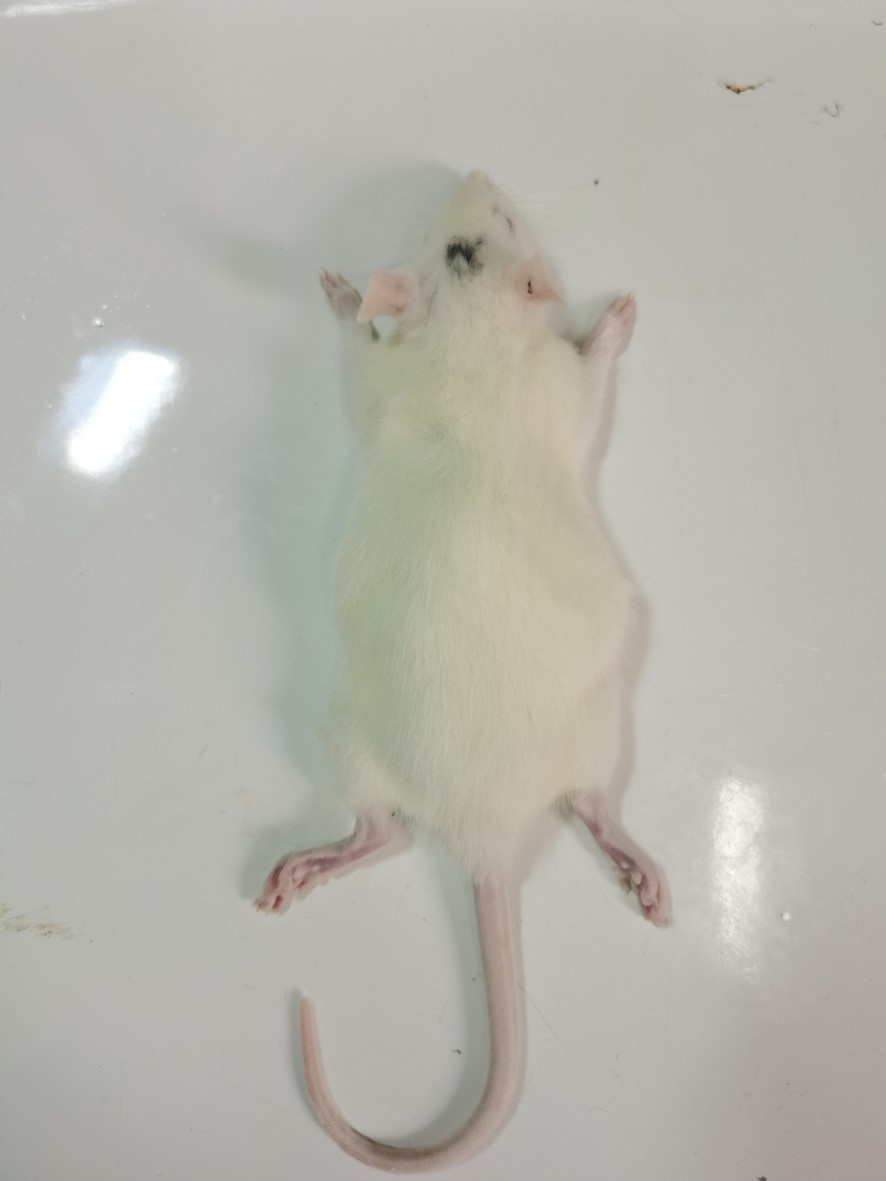

Supplement: Supplementary file 4 — Source Data [file 41467_2024_47559_MOESM4_ESM.zip › Figure 5d-uncropped photogragh.jpg]

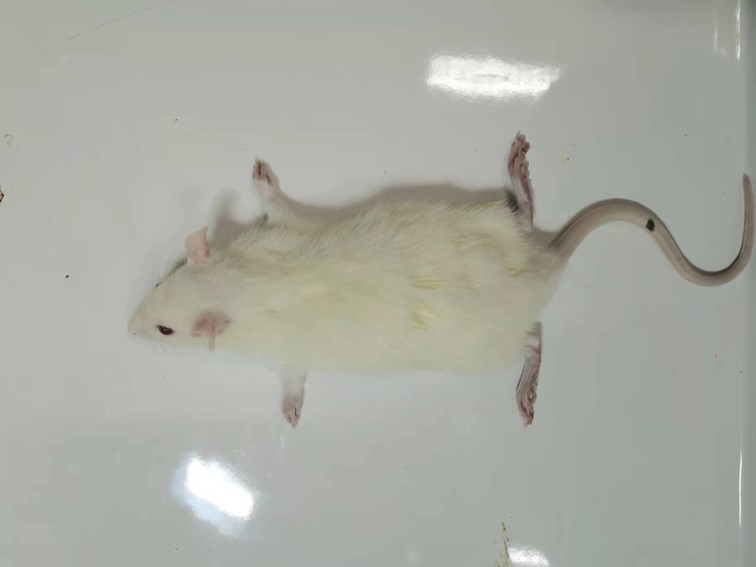

Supplement: Supplementary file 4 — Source Data [file 41467_2024_47559_MOESM4_ESM.zip › Figure 5e-uncropped photogragh.jpg]

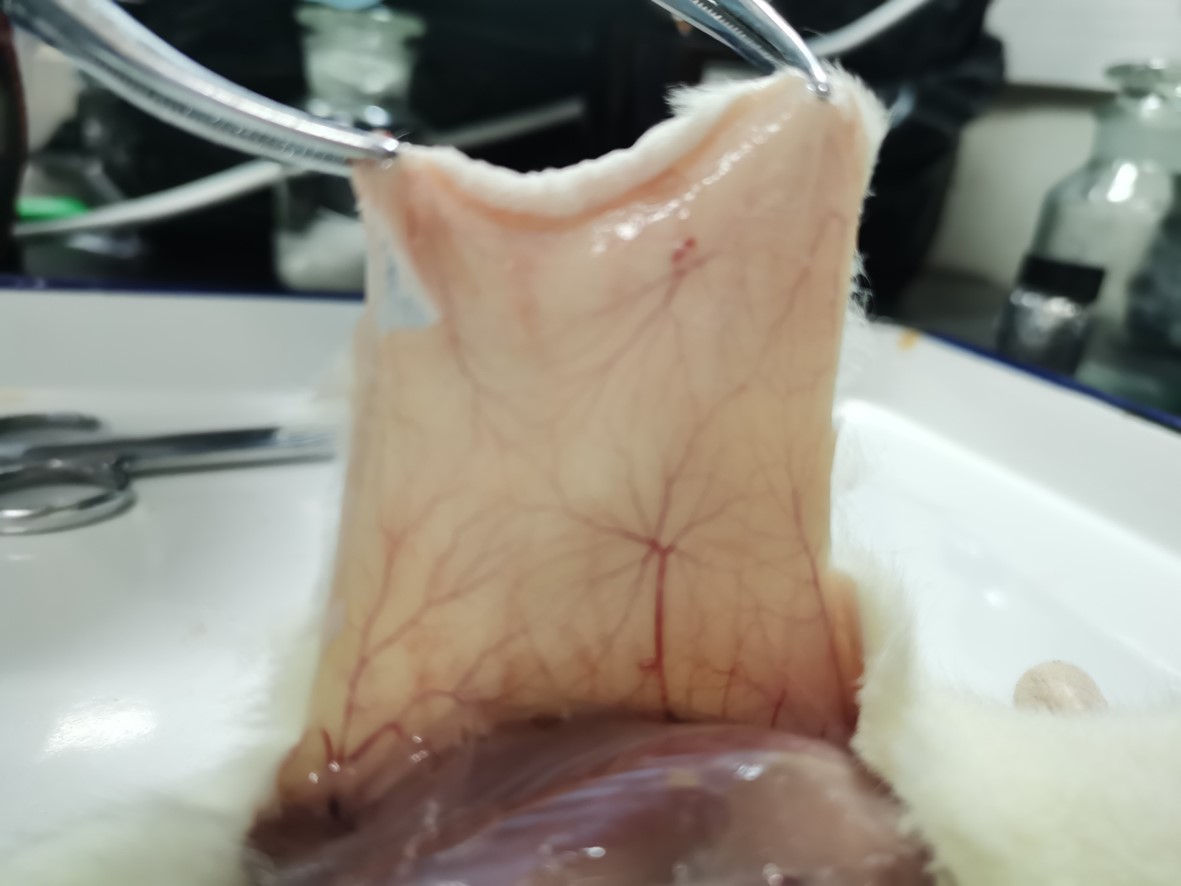

Supplement: Supplementary file 4 — Source Data [file 41467_2024_47559_MOESM4_ESM.zip › Figure 5f-uncropped photogragh.jpg]

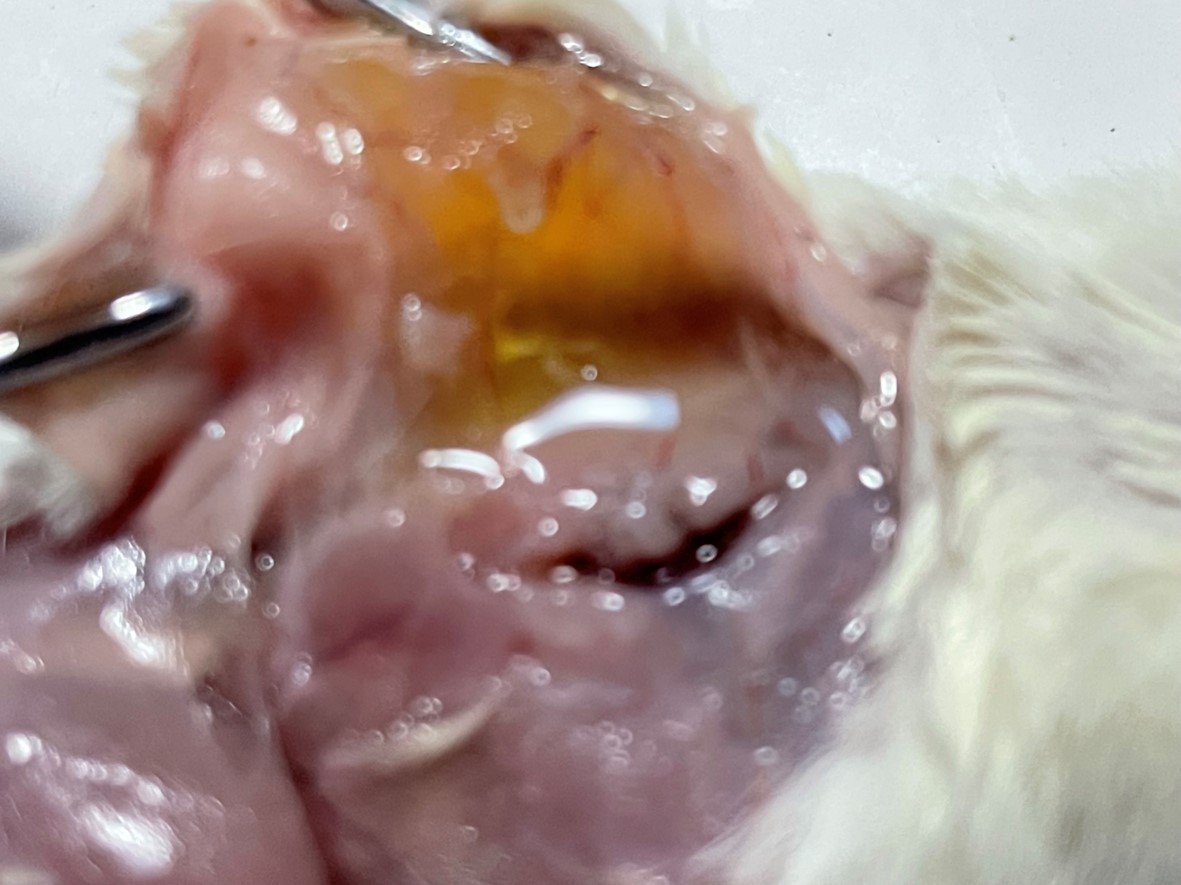

Supplement: Supplementary file 4 — Source Data [file 41467_2024_47559_MOESM4_ESM.zip › Figure 5g-uncropped photogragh.jpg]

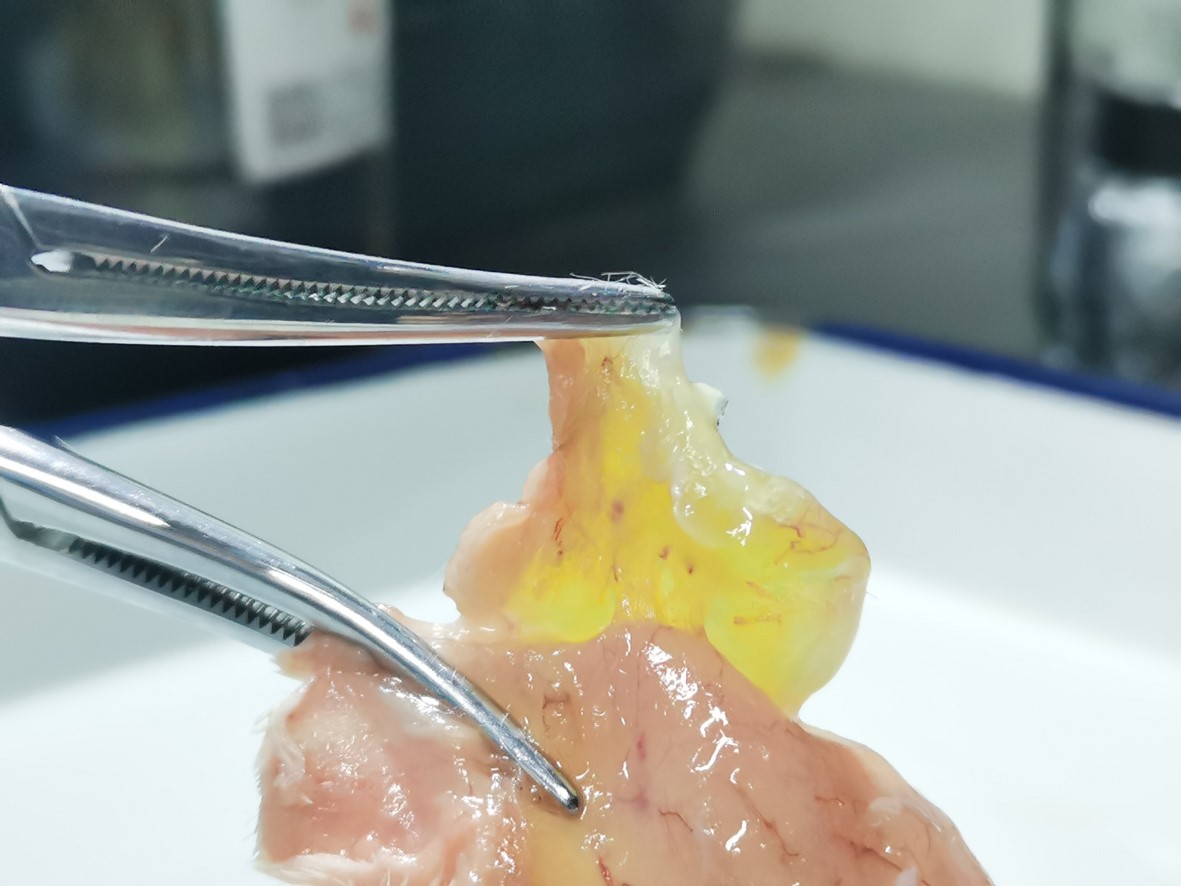

Supplement: Supplementary file 4 — Source Data [file 41467_2024_47559_MOESM4_ESM.zip › Figure 5h-uncropped photogragh.jpg]

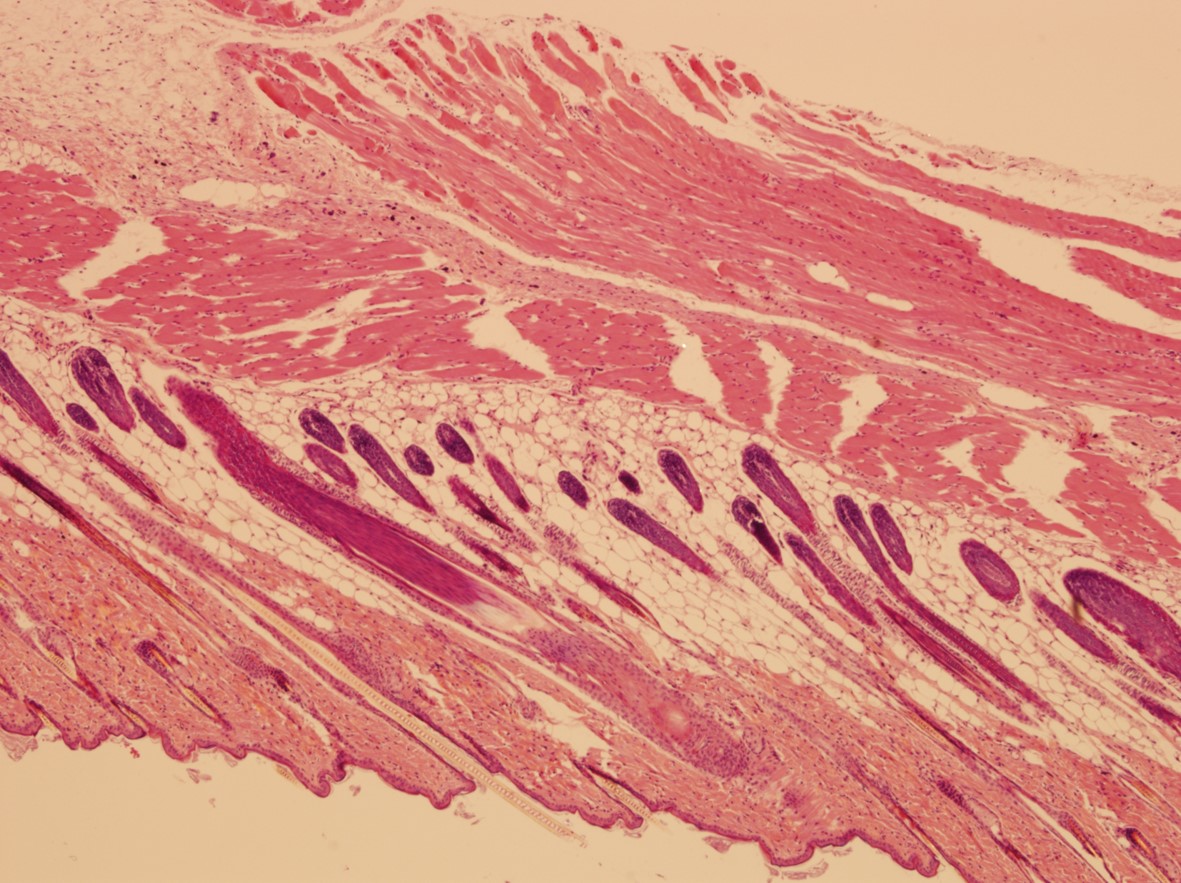

Supplement: Supplementary file 4 — Source Data [file 41467_2024_47559_MOESM4_ESM.zip › Figure 5i-uncropped photogragh.jpg]

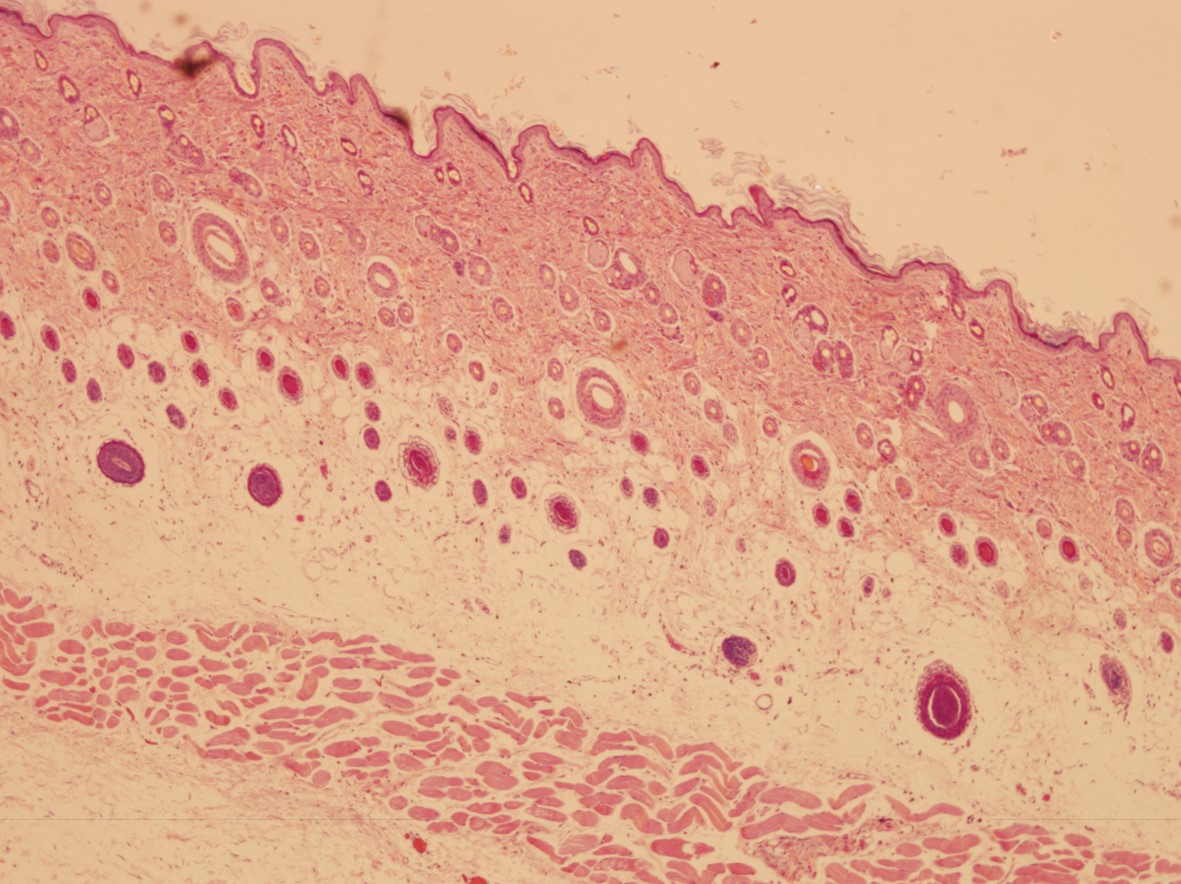

Supplement: Supplementary file 4 — Source Data [file 41467_2024_47559_MOESM4_ESM.zip › Figure 5j-uncropped photogragh.jpg]

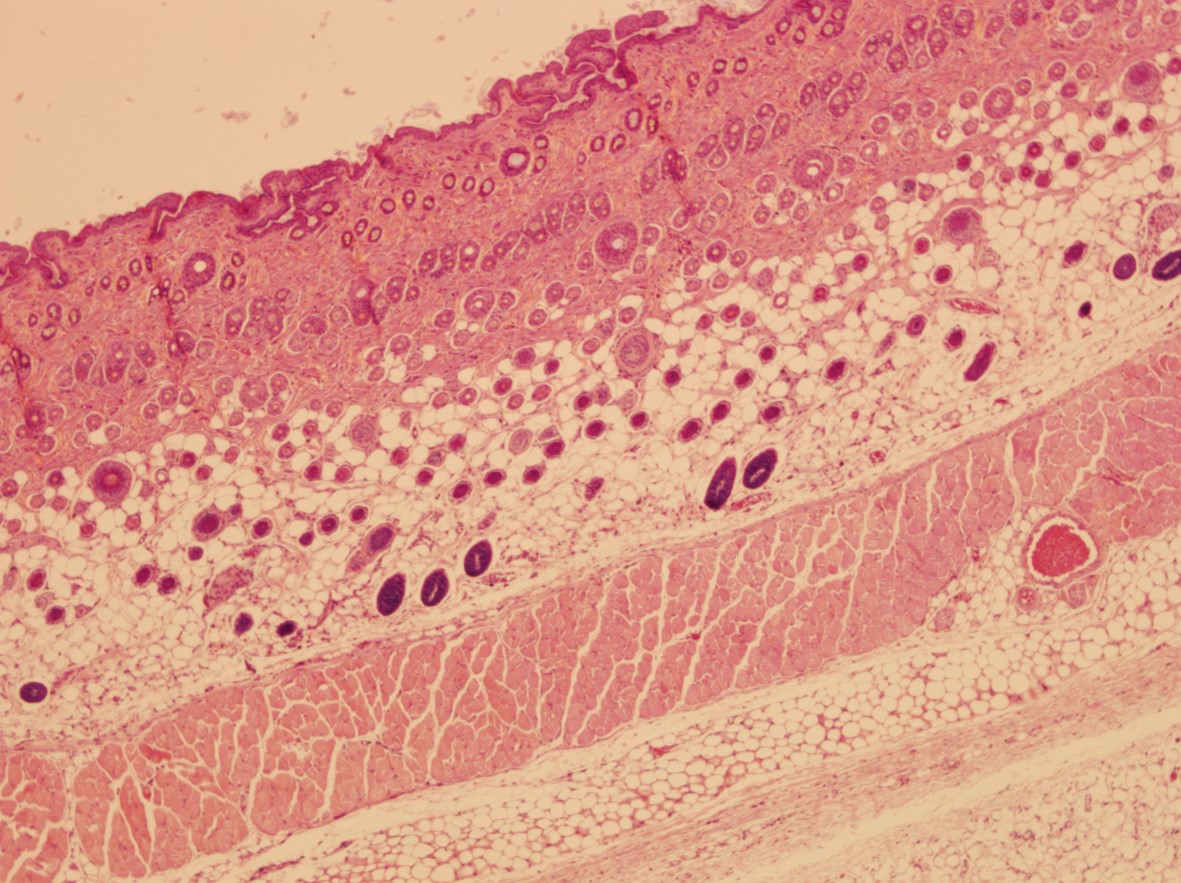

Supplement: Supplementary file 4 — Source Data [file 41467_2024_47559_MOESM4_ESM.zip › Figure 5k-uncropped photogragh.jpg]

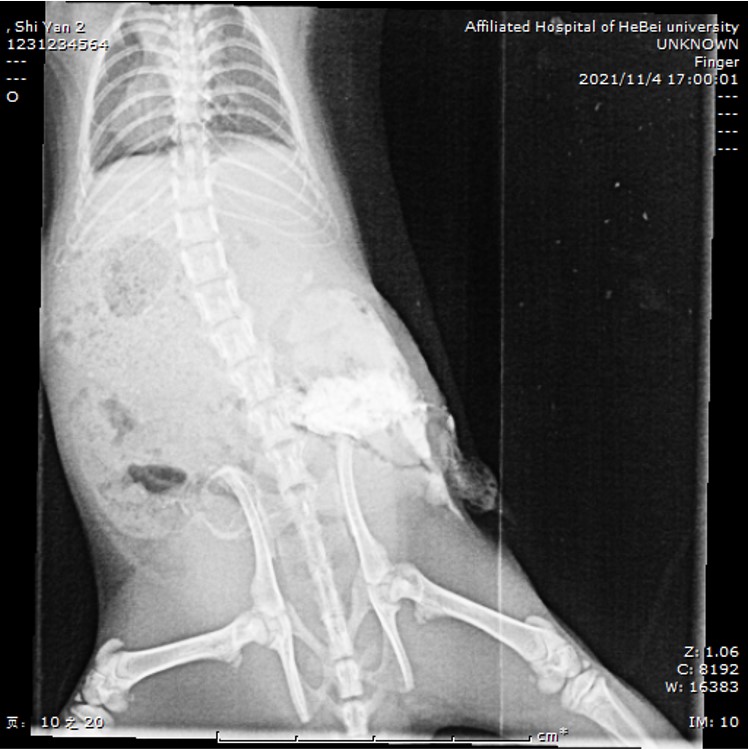

Supplement: Supplementary file 4 — Source Data [file 41467_2024_47559_MOESM4_ESM.zip › Figure 5l-uncropped photogragh.jpg]

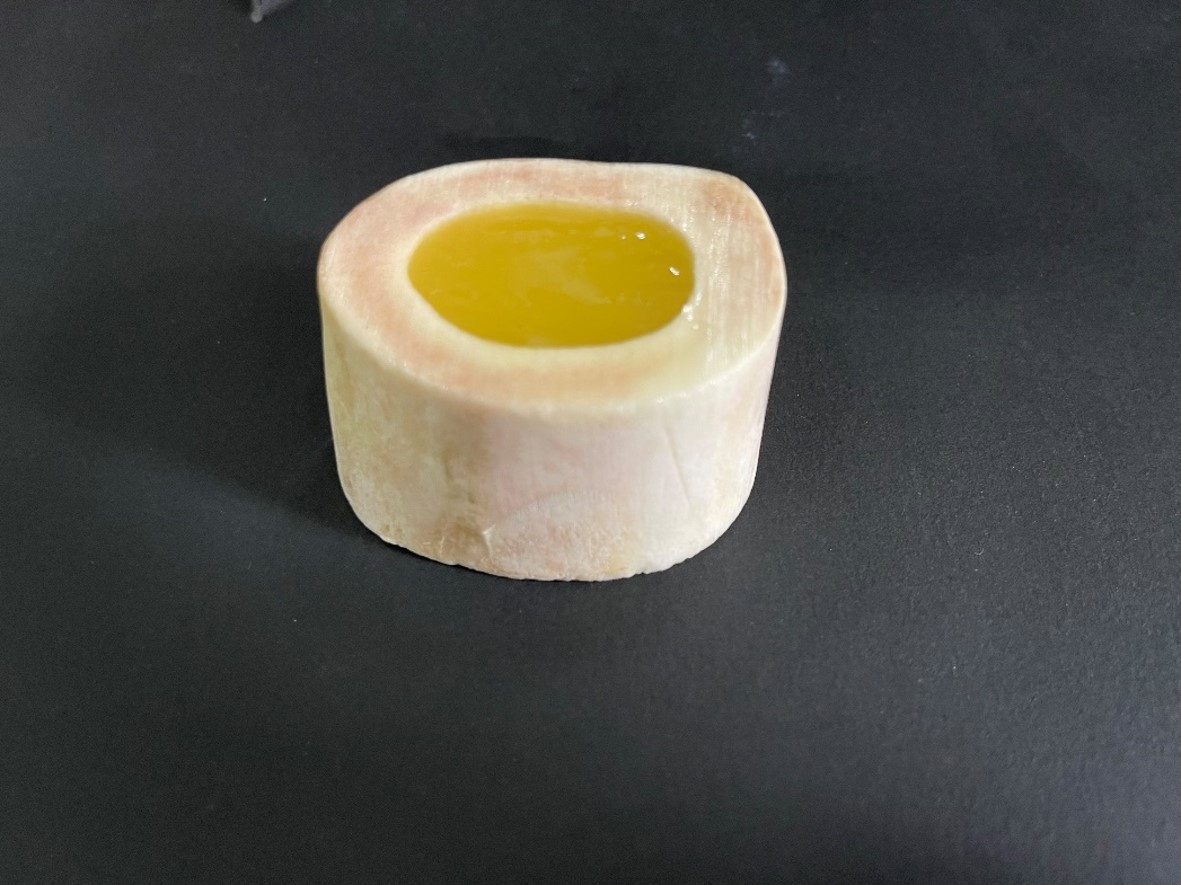

Supplement: Supplementary file 4 — Source Data [file 41467_2024_47559_MOESM4_ESM.zip › Figure 6b-uncropped photogragh.jpg]

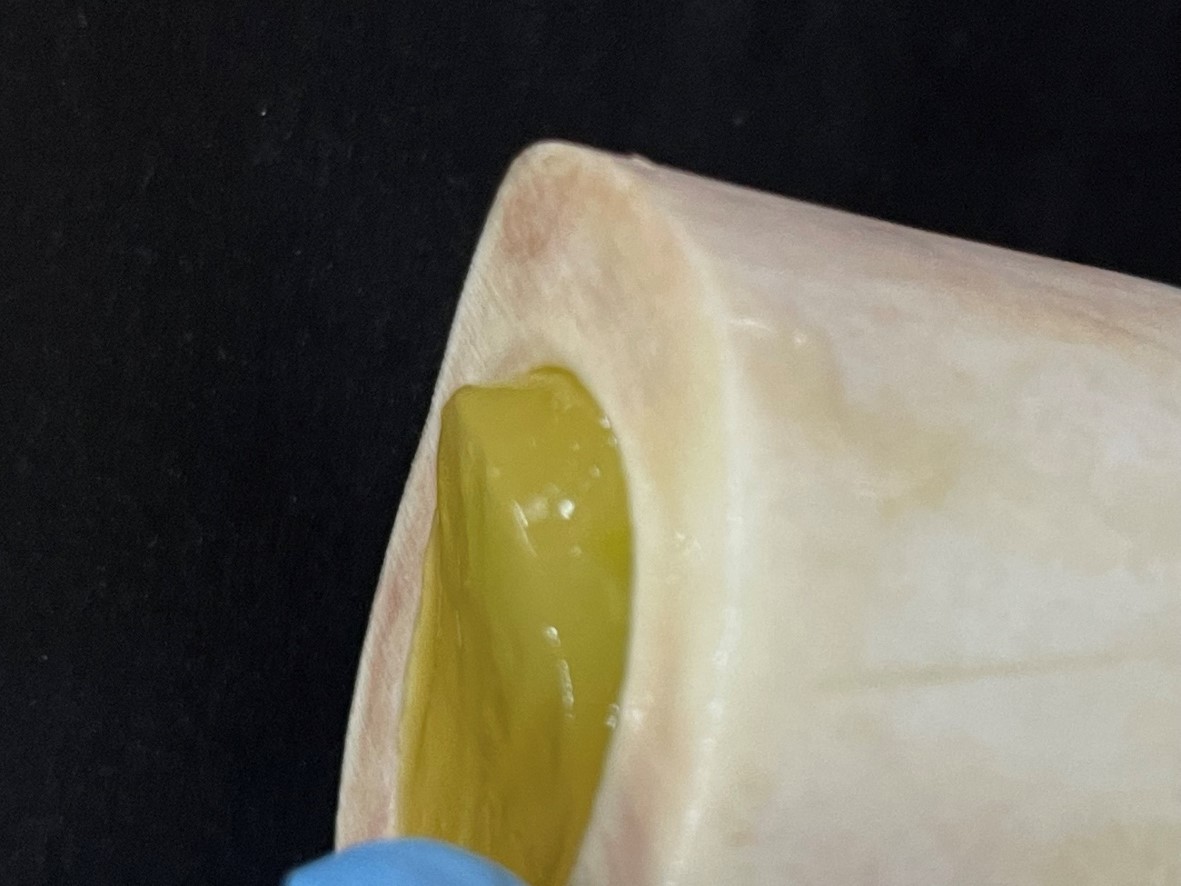

Supplement: Supplementary file 4 — Source Data [file 41467_2024_47559_MOESM4_ESM.zip › Figure 6c-uncropped photogragh.jpg]

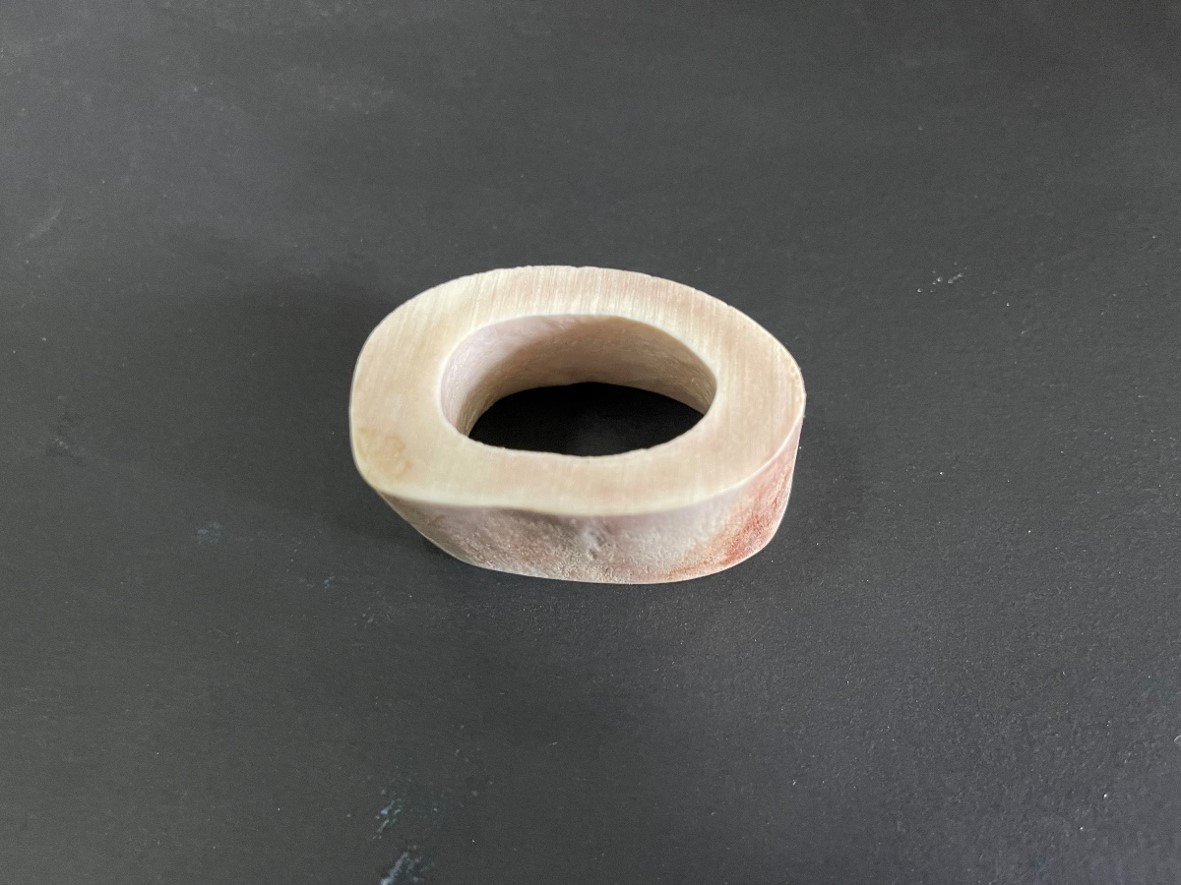

Supplement: Supplementary file 4 — Source Data [file 41467_2024_47559_MOESM4_ESM.zip › Figure 6d-uncropped photogragh.jpg]

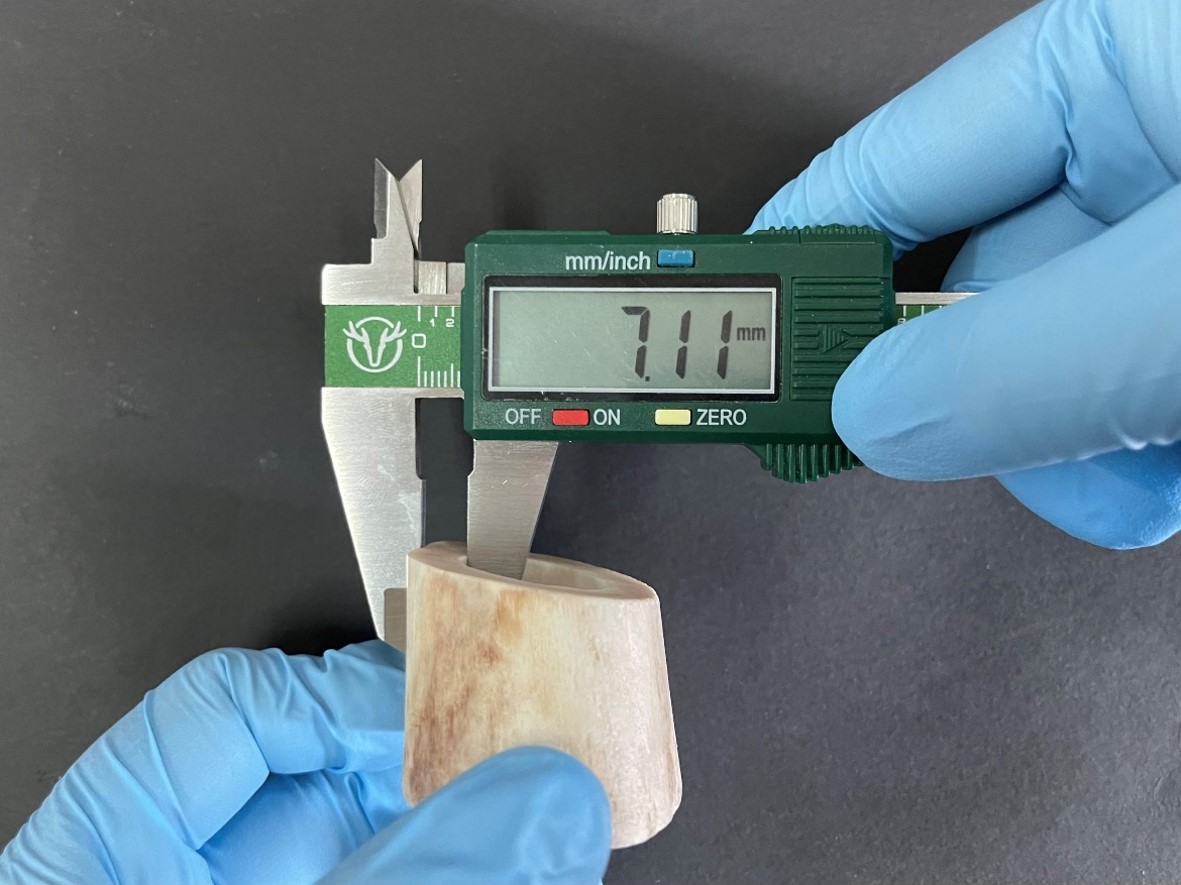

Supplement: Supplementary file 4 — Source Data [file 41467_2024_47559_MOESM4_ESM.zip › Figure 6f-uncropped photogragh.jpg]
